# Experimental charge density of grossular under pressure – a feasibility study

**DOI:** 10.1107/S2052252520001955

**Published:** 2020-03-07

**Authors:** Roman Gajda, Marcin Stachowicz, Anna Makal, Szymon Sutuła, Jan Parafiniuk, Pierre Fertey, Krzysztof Woźniak

**Affiliations:** a Biological and Chemical Research Centre, Department of Chemistry, University of Warsaw, Żwirki i Wigury 101, Warszawa 02-093, Poland; b Institute of Geochemistry, Mineralogy and Petrology, Department of Geology, University of Warsaw, Żwirki i Wigury 93, Warszawa 02-089, Poland; c Synchrotron SOLEIL, L’Orme des Merisiers - Saint Aubin, B.P. 48, Gif-sur-Yvette Cedex 91 192, France

**Keywords:** grossular, garnet, experimental charge density, high pressure, synchrotron radiation, multipole refinement

## Abstract

X-ray diffraction studies of crystals under pressure and quantitative experimental charge density analysis are among the most demanding types of crystallographic research. A successful feasibility study of the electron density in the mineral grossular under 1 GPa pressure conducted at the CRISTAL beamline at the SOLEIL synchrotron is presented in this work. A single crystal was placed in a diamond anvil cell, but owing to its special design (wide opening angle), short synchrotron wavelength and the high symmetry of the crystal, data with high completeness and high resolution were collected. This allowed refinement of a full multipole model of experimental electron distribution. Results are consistent with the benchmark measurement conducted without a diamond-anvil cell and also with the literature describing investigations of similar structures. Results of theoretical calculations of electron density distribution on the basis of dynamic structure factors mimic experimental findings very well. Such studies allow for laboratory simulations of processes which take place in the Earth’s mantle.

## Abstract 2

A successful feasibility study of experimental electron density in the mineral grossular under 1 GPa pressure was conducted at the CRISTAL beamline at the SOLEIL synchrotron. Such studies allow for laboratory simulations of processes that take place in the Earth’s mantle.

## 1. Introduction   

**Figure 1:**
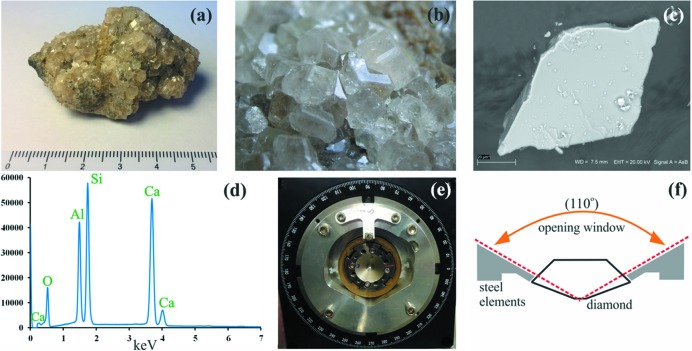
(*a*) Natural sample of grossular used in this study, from Bazhenowskoye, Ural Mts, Russia. Size of specimen single-crystal pieces: 14 × 10 × 10 mm. (*b*) Rare dodecahedral and hexaoctaedral faces developed on some crystals of this specimen. (*c*) Single crystal of grossular investigated by electron microscopy. (*d*) Magnified part of the spectrum showing the presence of ion substitutions. (*e*) Our DAC (Diacell One20DAC) mounted inside the Chi-circle with the use of an additional adapter (brownish element). (*f*) Schematic defining the opening window in a DAC.

In this work, we combine two technically demanding types of X-ray diffraction experiments: X-ray diffraction studies under pressure and experimental X-ray charge density analysis. A number of examples of experimental electron density determined for a crystal under high pressure have already been published. However, so far this has been achieved either for pure elements (Li *et al.*, 2015 ▸) or for inorganic compounds with the use of maximum entropy methods (Yamanaka *et al.*, 2009 ▸). An interesting attempt to apply the aspherical approach to study charge density in a molecular crystal structure (propionamide) under high pressure was presented as a poster at the ECA meeting (Fabbiani *et al.*, 2011 ▸). Recently, a description of such an attempt for the molecular organic crystal *syn*-1,6:8,13-bis­carbonyl­[14]annulene has also been published (Casati *et al.*, 2016 ▸), and certain challenges arising with charge density analysis in crystals at high pressure have been discussed by Casati and co-workers (Casati *et al.*, 2017 ▸). Obtaining experimental charge density distribution from high-pressure measurements is not trivial. The first problem is related to the use of diamond anvil cells (DACs) which have limited opening angles, thus leading to a poor completeness of the diffraction data. This is why we decided to investigate a mineral that crystallizes in the cubic system, for which the independent part of the reciprocal lattice is small. Moreover, we used a DAC with a much wider opening angle than in the cases of the most commonly used DACs (Fig. 1 ▸). Secondly, a much shorter wavelength available at the synchrotron facility made it possible to collect more reflections than in the case of an in-house X-ray source. Because the multipolar model required up to 34 parameters per atom, the number of collected reflections was very important. Thirdly, because the multipolar model developed by Hansen & Coppens (1978 ▸) has been known as imperfect in the description of heavier atoms (such as transition metals), we chose the model mineral grossular, consisting of relatively light elements.

Grossular, Ca_3_Al_2_(SiO_4_)_3_, is quite a common mineral member of the garnet group. It was first described by Abraham Gottlob Werner in 1803 as ‘cinnamon stone’ (‘Kanelstein’ in German). The name grossular (originally grossularite) was introduced by Werner in 1808 – describing the color and shape – for a mineral crystal from the Achtaranda River mouth in Vilui River Basin, Yakutia, now Sakha, Siberia, Russia. They are green and resemble gooseberry (Ribes grossularium) fruits. Pure grossular is colorless and almost transparent, but such specimens are very rare. Grossular crystallizes in the cubic space group 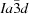 and is usually yellow to brown, but may also be orange, pink, red or green due to cation substitutions replacing calcium or aluminium in the mineral structure. The general formula for this group of minerals is *X*_3_ *Z*_2_(SiO_3_)_4_, where position *X* could be substituted by Fe^2+^, Mg^2+^, Mn^2+^ or Ca^2+^ cations and position *Z* by Al^3+^, Fe^3+^ or Cr^3+^. For more information on grossular and garnets see the supporting information. A piece of a single crystal of grossular investigated in this work was separated from a natural mineral sample (see Fig. 1 ▸) from the Bazenowskoje locality near Asbest town (Ural Mountains, Russia).

Because our sample was of natural origin, it contained traces of elements other than those present in the formula. We identified them by using the scanning electron microscope FE-SIGMA VP (Carl Zeiss Microscopy GmbH) with an energy-dispersive silicon drift detector (Quantax XFlash 6|10, Bruker Nano GmbH). Results of this measurement are presented in Fig. 1 ▸. A more detailed view of a scanning electron microscope energy-dispersive spectrum is provided in Fig. S1 of the supporting information. Analysis of different grains of grossular showed that, in addition to calcium, aluminium, silicon and oxygen, there are also traces of iron, barium, chromium and manganese. The measurement showed that the most significant substitution in this specimen was the substitution of iron, the concentration of which varied between 0.26 and 1.82%.

The main aim of our research was to obtain an experimental quantitative electron density distribution (EDD) for grossular on the basis of data collected under pressure (1 GPa) on a real mineral sample.

## 2. Methods   

### 2.1. Data collection   

The high-pressure experiment was carried out at the CRISTAL beamline at the SOLEIL synchrotron and was followed by measurement on an in-house diffractometer with a molybdenum microsource. The same piece of a single crystal of grossular separated from the natural sample was investigated in both experiments. The X-ray diffraction experiments were performed at 293 K with high resolution and completeness (100% completeness up to 0.45 Å).

#### 2.1.1. Synchrotron data collection   

Single-crystal data were collected using a dedicated experimental setup installed on the six-circle diffractometer, with two degrees of freedom for the sample orientation (*i.e.* the so-called Phi and Chi rotations whose axes are mutually perpendicular). A monochromatic beam was extracted from the U20 undulator beam by means of an Si(111) double-crystal monochromator (DCM). The beam was shaped to a beam size of 50 × 50 µm (full width at half-maximum) at the sample position, using focalization optics (sagittal focusing with the second crystal of the DCM, vertical focusing using Pt-coated Si mirrors) and slits. Two-dimensional images were collected with a RayoniX SX-165 CCD detector.

The sample was placed in a diamond-anvil cell (DAC) equipped with 0.5 mm culet diamonds and fitted with a steel gasket of initial thickness 0.2 mm and a 0.3 mm gasket hole. The grossular crystal was placed inside together with a small ruby sphere for reference pressure measurements. Paratone oil was used as a pressure-transmitting medium. External pressure (*ca* 1 GPa) was applied and verified by the ruby reference.

The particular DAC (DiacellOne20DAC – see Fig. 1 ▸) used in this experiment had an effective opening angle equal to 110°, according to the manufacturer Almax easyLab. The goniometer at the CRISTAL beamline is equipped with a Chi circle, which accommodates membrane DACs with a diameter equal to 60 mm. A dedicated adapter was 3D-printed to fit the 49 mm diameter of our DAC (see Fig. 1 ▸). As a consequence of hardware constraints at CRISTAL (*i.e.* a bulky Chi circle), the effective opening angle of our DAC was reduced to 98°. Nevertheless, the orientation of the sample could be varied owing to the Chi rotation axis, effectively increasing the attainable data coverage. The Chi circle could rotate about 360° and the detector (CCD camera) could be rotated up to 40° with respect to the X-ray beam direction. Image acquisition consisted of a succession of rotating images using the phi rotation (*i.e.* 1° rotation of the sample per image, 1 s exposure time) over a 98° angular range. We collected data at three different Chi circle positions and three different detector positions. Some of the runs were collected with and without an attenuator to make sure that weak reflections were all recorded while strong reflections were not overexposed. The attenuator used decreased the intensity of the beam *ca* 5 times. The exact high-pressure data collection strategy is defined in Table S5 of the supporting information.

#### 2.1.2. Synchrotron wavelength calibration   

Data collection was performed on a reference sample (ruby NIST SRM 1990) to calibrate the wavelength of the monochromatic beam. A 180° rotation scan (1° per image) was acquired at the 0° detector position, completed by three rotation scans of 90° each with the detector position set at 15, 30 and 40° with respect to the beam direction. The images were processed with the *CrysAlis PRO* software suite (Rigaku Oxford Diffraction, 2015 ▸). The goniometer parameters were refined, keeping the cell parameters fixed at the referenced NIST values. New cell parameters were recalculated at the end of the refinement process: the wavelength was then adjusted until the final calculated cell parameters were within one standard deviation with respect to the NIST values. The wavelength was thus determined to have a value of 0.4166 Å.

#### 2.1.3. Sample centering at the synchrotron   

Once the DAC was mounted on the goniometer and roughly centered with the help of an optical microscope, a fine centering was performed using the X-ray beam. Firstly, short scans of the DAC, perpendicular to the direction of the beam (*i.e.* horizontal *y* and vertical *z* directions) were performed, while the intensity of the X-ray beam coming through the gasket hole was recorded with a silicon diode. The position of the gasket center was deduced as the geometric center of the resulting plots of the X-ray intensity versus translation. This way, the geometric center of the gasket could be placed at the goniometer center.

In the case of the X-ray beam direction (*x*), the adjustment of the DAC was realized using two scans along *y* performed consecutively, with the DAC rotated a few degrees with respect to the X-ray beam direction clockwise and counter-clockwise. The sample translation along *x* was then adjusted until the intensity versus translation plots registered at both positive and negative inclinations overlapped perfectly.

Once the center of the gasket hole was perfectly positioned at the center of the goniometer, the second and final stages of sample centering were performed. Since the position of the beam on the images of the microscope used to visualize the sample was identified, for each Chi value, the DAC was translated in the *z* and *y* directions until the actual crystal was superimposed onto this known position, ensuring that the sample stays at the beam position during the whole data collection.

However, the sample would only be well centered along the X-ray beam if it was located in the middle of the pressure chamber delimited by the gasket and the diamond inner faces of the DAC. If a thin crystal was placed on one of the culets, its center would remain shifted with respect to the goniometer center by about half of the gasket thickness. Such sample misalignment would affect the true sample-to-detector distance, and hence the experimentally derived unit-cell parameter. Indeed, simple calculations based on Bragg’s equation for grossular off-centered 100 µm towards the detector (*i.e.* shortening the crystal-to-detector distance by half the thickness of the gasket used) would result in shortening of the unit-cell parameter by 0.01 Å. For the data collected without the DAC, from several detector positions, such miss-centering can be eliminated at the data-reduction stage owing to refinements of global unit-cell parameters and the instrument model. In the case of high-pressure measurements with a limited number of reflections that could be recorded due to the DAC environment restrictions, such misalignment cannot be corrected and may partly account for the slightly larger unit-cell parameter observed at 1 GPa.

#### 2.1.4. In-house data collection   

The in-house data collection was conducted at ambient temperature and pressure and was used as a benchmark for the synchrotron experiment. A grossular specimen was mounted on top of a thin glass capillary with a tiny amount of ep­oxy resin. An optimal data collection strategy, yielding a complete dataset up to the above resolution was calculated and executed with the *CrysAlis PRO* software (Rigaku Oxford Diffraction, 2015 ▸).

### 2.2. Data reduction   

Data reduction from all the frames collected was processed using the *CrysAlis PRO* software (Rigaku Oxford Diffraction, 2015 ▸). The reflections inherent to the diamond crystals of the DAC were indexed and omitted in data processing. For each of the two measurements (*i.e.* synchrotron and laboratory experiments), the resolution of the data was restricted to 0.45 Å to maintain full completeness. Next, the structures were solved and refined with *ShelXS* (Sheldrick, 2008 ▸) and *ShelXL* (Sheldrick, 2015 ▸), respectively, within the *Olex2* suite (Dolomanov *et al.*, 2009 ▸). Then, the intensities for each of the measurements were merged using *Sortav* (Blessing, 1995 ▸) implemented in the *WinGX* program suite (Farrugia, 2012 ▸). Such merged intensity data were subsequently used as input for the program *XD2016* (Volkov *et al.*, 2016 ▸). The quality of the *hkl* datasets obtained is characterized in the supporting information.

### 2.3. Multipole refinements   

The structure of grossular solved and refined at the independent atom model level using *SHELX*-97 (Sheldrick, 2008 ▸) served as a starting point for the refinement of the Hansen–Coppens multipole model. Refinements against the data collected at SOLEIL (herein denoted Exp_1GPa) and the data collected in-house (denoted Exp_Amb) proceeded in the same way. The refinement was conducted on *F* ^2^, and weighting parameters refined by *ShelXL* were used.

In both cases, the model included a multipole expansion up to the *l* = 4 level (hexadecapoles). Because Ca, Si and Al atoms occupy special positions, only certain symmetry-allowed multipoles were taken into account. Moreover, special constraints, which allow us to present normalized cubic harmonics as linear combinations of spherical harmonics, were used. Each model was refined in 13 steps. Details of these steps and their order are reported in the supporting information. The scale factor was refined in each step. No significant correlation between the refined parameters was observed.

### 2.4. Theoretical calculations   

Calculations of theoretical electron density distribution were conducted using *CRYSTAL17* software (Dovesi *et al.*, 2017 ▸, 2018 ▸) in which the crystal structure of grossular was optimized. Optimizations of the structure at 1 GPa and at ambient pressure were first realized with the unit-cell parameter fixed at the experimental values (variants 1 and 2, respectively). Three other variants were simulated, for which the structure and the unit-cell parameter were optimized at ambient pressure (variant 3), 1 GPa (variant 4) and 10 GPa (variant 5).

The charge density distributions of these variants were characterized using two different approaches. In the first approach, theoretical dynamic structure factors were calculated with *CRYSTAL17* (Erba *et al.*, 2013 ▸) and then used to refine a multipolar model of the electron density using the program *XD2016*, according to the same procedure used in the case of the experimental data. The refinement corresponding to the five structure variants described above will be referred to as *theoretical refinement i* and labeled TR*i* (*i* = 1–5) in the following sections.

In the second approach, topological analysis of the theoretical charge density distributions was performed with the program *TOPOND14* (Gatti *et al.*, 1994 ▸; Gatti & Casassa, 2013 ▸) already implemented in the *CRYSTAL17* package.

These theoretical calculation results were used to benchmark both the experimental results obtained from the synchrotron measurement and the in-house diffractometer. Furthermore, they give relevant insights into how properties at bond/interaction critical points (BCPs) depend on the value of the *a* parameter we used in the different structure optimization variants.

## 3. Results and discussion   

### 3.1. Crystal structure   

**Figure 2:**
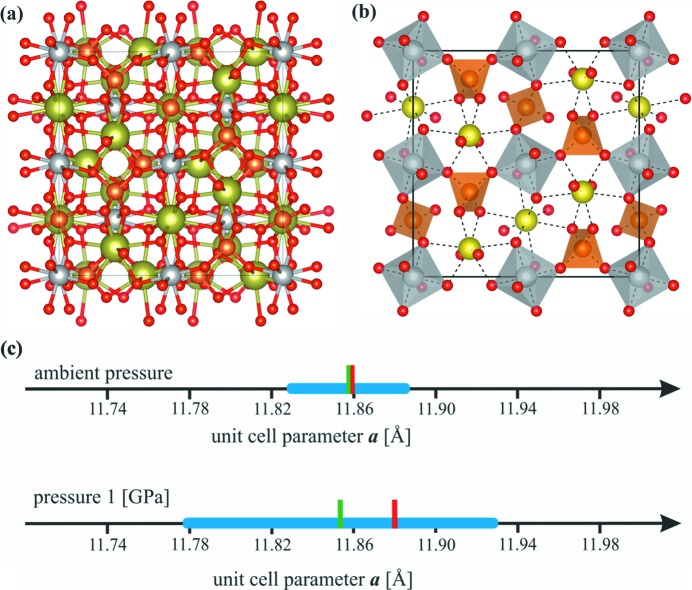
(*a*) Unit-cell contents of grossular (Ca: yellow balls, Al: gray balls, Si: orange balls, and O: red balls). (*b*) Graphical representation of the first-layer AlO_6_ and SiO_4_ polyhedra in the grossular crystal structure. (*c*) The thick horizontal blue bar illustrates confidence intervals (the average value ±3 sample standard deviations) of the parameter *a* at ambient conditions (the upper line) and 1 GPa (the bottom line). The thin-green vertical bars indicate the average value of the *a* parameter of ten measurements. The thin red vertical bars indicate the particular values for Exp_Amb (upper part) and Exp_1GPa (lower part).

Selected details of data collections and refinements are presented in Table 1 ▸. Several results from this table deserve closer inspection. Let us mention the different absorption corrections used in both cases. The DAC prevented the use of more advanced absorption correction procedures in the case of synchrotron data collection. Although the unit-cell parameter *a* determined at 1 GPa appeared slightly larger than expected based on the laws of thermodynamics, it was still within the margin of experimental error (see Fig. 2 ▸ *c*). More information about the estimation of the experimental standard deviation for this parameter as well as other details regarding the quality of the measured data are reported in the supporting information.

Grossular, Ca_3_Al_2_(SiO_3_)_4_, exhibits a centered cubic symmetry (space group 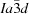). It has pseudo-dodecahedral sites (Ca, 24*c* [Wyckoff notation], 2.22 [site symmetry]), pseudo-octahedral sites (Al, 16*a*, −3) and pseudo-tetrahedral sites (Si, 24*d*, −4). Calcium ions are placed at an intersection of three twofold rotation axes, silicon ions at a fourfold rotoinversion point and aluminium ions on a threefold rotoinversion center (see Fig. 2 ▸). There is only 1/4 Ca and Si atoms and 1/6 Al atoms in the asymmetric part of the unit cell. Each oxygen atom is bonded to one Si atom, one Al atom and two Ca atoms in a highly distorted tetrahedral configuration. An extensive description of crystal chemistry across the garnet group, including some systematic trends, was given by Antao (2013 ▸). The unit cell of grossular is presented in Fig. 2 ▸.

### 3.2. Structural parameters and ADPs   

A comparison of interionic distances determined from the experimental and theoretical models is given in Table 2 ▸. When one considers the distances obtained experimentally, all the values obtained from data collected at 1 GPa seem to be slightly longer than those obtained at ambient pressure. This is probably caused by the combination of the slightly longer *a* parameter measured and negligible structural effects exerted by a relatively small 1 GPa applied pressure. However, from a careful comparison of the differences calculated between the experimental values at ambient pressure (Exp_Amb) and the theoretical values at the same pressure (TR3) on the one hand, and the differences observed between the experimental values at 1 GPa (Exp_1GPa) and ambient pressure (Exp_Amb) on the other hand, one can conclude that the slightly higher interatomic distances at 1 GPa, with respect to those at ambient pressure, are meaningless. As demonstrated by the theoretical evolution of these interionic contacts as a function of the applied pressure, one can see that changes in distance are perfectly consistent with the shrinking of the *a* parameter. Note that a 10 GPa variation (comparison of TR4 and TR5) induces a contraction of the interatomic distances of about 1.5%.

Atomic displacement parameters (ADPs) for the ions forming the grossular structure obtained experimentally are shown in Table 3 ▸. ADPs obtained using the theoretical dynamic structure factors are presented in Table S6 of the supporting information.

When we compare the experimental results, with the exception of Al, the ADP values of other ions are larger under 1 GPa pressure (synchrotron measurement) than those obtained for data collected at ambient pressure (in-house diffractometer). For Al the ADPs at 1 GPa are smaller than those for Al at ambient conditions, although all differences are rather small.

Here also, when considering the results obtained by theoretical calculations and the differences between experimental and theoretical values as discussed for the interatomic distances, no significant differences are observed between the 1 GPa and ambient-pressure ADPs values.

### 3.3. Topology of experimental charge density distribution   

One of the most obvious indicators of the similarity between Exp_Amb and Exp_1GPa are properties of the charge density at BCPs. In the case of grossular, we have only three types of interatomic contacts (Ca—O, Al—O, Si—O) and four different BCPs (two different Ca—O contacts).

Information about distances between atoms and BCPs, electron density at BCPs and the corresponding Laplacian for data obtained experimentally as well as results of theoretical calculations are summarized in Table 4 ▸. Additional information about experimental data, including the Hessian tensor diagonal values λ_1,_ λ_2,_ λ_3_ and ellipticity, can be found in Table S4 of the supporting information. The parameters reported in Tables 4 ▸ and S4 for the topological analysis of the experimental charge densities at ambient pressure and 1 GPa are in agreement with an expected marginal effect of the pressure for a compound with a room-pressure bulk modulus of about 150 GPa (Hansen & Coppens, 1978 ▸).

First of all, it is worth considering whether or not properties at BCPs for data obtained experimentally are comparable with those obtained theoretically and vice versa. Of course, one has to remember that each refinement is characterized by a slightly different value of the unit-cell parameter, which can affect the properties considered. The value of the *a* parameter in the case of TR1 and TR2 is equal to the value obtained experimentally for synchrotron and laboratory measurements, respectively. For theoretical refinements 3, 4 and 5, the *a* parameters were freely refined and achieved the values 11.82951 (10), 11.80797 (10) and 11.63202 (10) Å, respectively.

When comparing the distances from particular ions to the corresponding BCPs (*d*_1-bcp_ and *d*_2-bcp_ in Table S4), one can see that theoretical values at ambient pressure and at 1 GPa are quite consistent. The values hardly shrink in accordance with the shrinking of the unit-cell parameter and mimic the experimental values quite well. However, the results from *XD2016* are not identical to those from *TOPOND*. There seems to be a small systematic positive difference between the *d*_1-bcp_ values obtained with *XD2016* and *TOPOND*. Of course for *d*_2-bcp_ the relation is the opposite. The best agreement between the experimental and theoretical parameters is observed for the values of the electron density at BCPs (ρ(*r*_c_) in Table 4 ▸). In the case of Al—O, all the theoretical values are consistent when compared with the experimental ones, but underestimated by about 0.1 e Å^−3^. However, Al—O is not the best example to judge agreement of theoretical calculations with experimental results. This is because Al is partly substituted by Fe (discussed in the Introduction[Sec sec1]).

On the other hand, for Ca—O^I^, both *XD* and *TOPOND* slightly overestimate the values of the density, whereas for Ca—O^II^, both types of calculations correspond with experimental results quite well.

More significant are the differences between theoretical and experimental values of the Laplacian (∇^2^ρ(*r*_c_) in Table 4 ▸), which, as the second derivative of the charge density, is very sensitive to a change. In fact, results are consistent only in the case of the Ca—O^I^ interaction. For Ca—O^II^, both types of theoretical calculations give slightly higher values of the Laplacian than the experimental ones. For Si—O, when considering the discrepancies between the results obtained from *XD* and *TOPOND*, no clear conclusion can be drawn concerning the comparison with the experimental results. It is interesting however to notice that theoretical values of the Laplacian obtained from *TOPOND* are almost identical to the literature data for isostructural pyrope (Destro *et al.*, 2017 ▸) and closer to the experimental values determined in this work. In the case of Al—O, both theoretical calculations are discrepant from each other, but both give higher values for the Laplacian than those obtained from the experiments. To conclude this part, it seems that calculations are generally in good agreement with the experiments, at least in the case of charge density at BCPs, but the results for the Laplacian are much less consistent.

The next step is to compare results of our experiments with literature data. Because experimental and theoretical electron densities have been already determined for quite a few minerals, there are literature references of interest showing values of charge density and its Laplacian at BCPs. For example, the charge density topology of pyroxenes described by many authors between 1969 and 2001 was discussed by Downs (2003 ▸) and can work in this comparison.

However, the most relevant example of EDD described in the literature concerns pyrope (Destro *et al.*, 2017 ▸), which is isostructural with grossular (pyrope in Table 4 ▸). However, data for pyrope were collected at 30 K and the multipole model was developed up to the tricontadipoles (hexadecapoles in this work) and the calcium atom was substituted by a magnesium atom. Moreover, Destro and co-workers also used ADPs augmented by Gram–Charlier expansion with coefficients up to the third order for Si and O, and up to the fourth order for Mg and Al, whereas we used only the second-order ADPs for each atom type. Still, there is excellent agreement between the above datasets. In the case of Ca—O/Mg—O contacts, it is difficult to compare them as they involve completely different elements. Surprisingly, the values of electron density, Laplacian or energy densities seem to be quite similar. This may result from the fact that both atoms contribute two valence electrons and are quite alike in terms of chemical properties.

Aside from the case of pyrope, we can also look into some other examples. In the case of properties at the BCP for the Si—O bond, the literature is quite rich because many silicates have been investigated so far. As the values of charge density ρ and their Laplacian ∇^2^ρ depend on bond length, studies which take this into account are very informative (Gibbs *et al.*, 2005 ▸, 2014 ▸). Considering that, at ambient pressure, the Si—O bond distance in grossular is 1.6466 (2) Å (in-house experiment), on the basis of the mentioned literature, the values of the charge density ρ and the Laplacian ∇^2^ρ are expected to be around 0.88 e Å^−3^ and 18.5 e Å^−5^, respectively. The results obtained in our study are different, especially for the Laplacian. The values of ρ and ∇^2^ρ are equal to 1.15 e Å^−3^ and 8.5 e Å^−5^, respectively (see Table 4 ▸). The value of the Laplacian is therefore *ca* 50% smaller than expected.

For Al—O and Ca—O bonds, there is literature describing the electron density distribution in minerals such as topaz, Al_2_(SiO_4_)F_2_ (Ivanov *et al.*, 1998 ▸); α-spodumene, LiAl(SiO_3_)_2_ (Kuntzinger & Ghermani, 1999 ▸; Prencipe *et al.*, 2003 ▸); natrolite, Na_2_(Al_2_Si_3_O_10_)·2H_2_O; mesolite, Na_2_Ca_2_(Al_2_Si_3_O_10_)_3_·8H_2_O; scolecite, Ca(Al_2_Si_3_O_10_)·3H_2_O (Kirfel & Gibbs, 2000 ▸); danburite, CaB_2_Si_2_O_8_ (Luaña *et al.*, 2003 ▸); diopside, CaMgSi_2_O_6_ (Bianchi *et al.*, 2005*a* ▸); datolite, Ca[BOH(SiO_4_)] (Ivanov & Belokoneva, 2007 ▸); and clinopyroxene, LiGaSi_2_O_6_ (Bianchi *et al.*, 2005*b* ▸).

When we look at values of ρ and ∇^2^ρ at BCPs for the Al—O contacts in the above mentioned minerals, it is clear that the closest crystal environment has a significant influence on these parameters, and discrepancies are noticeable. The average values of ρ and ∇^2^ρ are equal to 0.658 e Å^−3^ and 14.81 e Å^−5^ (natrolite), 0.71 e Å^−3^ and 10.76 e Å^−5^ (mesolite), 0.67 e Å^−3^ and 12.31 e Å^−5^ (scolecite), or 0.64 e Å^−3^ and 4.78 e Å^−5^ (topaz). In our studies of grossular, we observe ρ = 0.515 e Å^−3^ and ∇^2^ρ = 1.394 e Å^−5^ (see Table 4 ▸).

When we take into consideration the properties at BCPs of Ca—O, discrepancies are also significant. Experimental values of ρ vary between 0.142 (3) e Å^−3^ and 0.184 (5) e Å^−3^ (danburite), 0.11 (1) e Å^−3^ and 0.26 (1) e Å^−3^ (diopside), and 0.114 e Å^−3^ and 0.360 e Å^−3^ (datolite). In mesolite and scolecite, the average value of ρ is equal to 0.226 e Å^−3^. As a result, the values of the Laplacian also vary from 3.23 e Å^−5^ to 4.14 e Å^−5^ (danburite), 1.9 (1) e Å^−5^ to 4.8 (1) e Å^−5^ (diopside), and 1.81 e Å^−5^ to 5.47 e Å^−5^ (datolite). In mesolite and scolecite, the average value of the Laplacian is equal to 3.57 e Å^−5^. For grossular (in this study), at two BCPs describing the Ca—O contacts, the values of ρ and ∇^2^ρ are as follows: 0.192 e Å^−3^ and 2.979 e Å^−5^, and 0.275 e Å^−3^ and 4.785 e Å^−5^, which are in very good agreement with the literature data.

Another issue to consider is the potential differences in the integrated properties of the electron density obtained by performing atomic basin integration. Details about atomic volumes and net atomic charge obtained from atomic basin integration conducted in *TOPXD* (both on experimental and theoretical data) are presented in Table 5 ▸.

When one takes into consideration the net atomic charge for ions (both experimental and theoretical results), we see some differences. Comparison of Exp_1GPa and TR1 (same *a* parameter) shows the most significant differences in the case of the Al cation (0.46 e); for other ions the difference is much smaller. Comparison of Exp_Amb and TR2 (the same *a* parameter) also shows a significant difference (0.72 e) in the case of the Al cation, but in the case of the Si cation it is even larger (0.81 e). However, one must remember that, in this natural piece of grossular, Al is partly substituted by Fe (0.26 to 1.82%) which is why all figures devoted to Al could be slightly biased.

It must be stressed that although theoretical calculations correspond very well with Exp_1GPa, Exp_Amb deviates from Exp_1GPa as well as from theoretical calculations. The sum of charges per molecule defined as Ca_3_Al_2_Si_3_O_12_ is equal to −0.06 e and +0.13 e for the ambient conditions and 1 GPa, respectively. For the corresponding theoretical calculations, this sum of charges is equal to +0.24 e for TR1 and TR2.

The largest values of Lagrangians were observed for silicon ions: 3.08 × 10^−3^ Ha and 2.56 × 10^−3^ Ha under ambient conditions and 1 GPa, respectively. One should remember that, for molecular crystals such as oxalic acid, sample standard deviations (SSD) for integrated atomic volumes and atomic charges range from 0.1 to 0.3 Å^3^ and from 0.02 to 0.10 e Å^−3^, respectively, so the discrepancies from neutrality, as well as the observed effects of pressure for the integrated properties are within +/−1 SSD (Kamiński *et al.*, 2014 ▸).

For the atomic volumes, the most significant differences between experiment and theory are those observed for the Si atomic basin. The difference between Exp_1GPa and TR1 is 1.08 Å^3^ (32% towards the theoretical value), and between Exp_Amb and TR2 is 2.24 Å^3^ (66% towards the theoretical value). The smallest differences are observed for the atomic basin of the Ca cation and oxygen anion. Again, Exp_Amb deviates from Exp_1GPa as well as from theoretical calculations

Integrated atomic volumes for Si and Al in Exp_Amb are larger than expected. On the other hand, they are very consistent with the results of experimental density analysis for pyrope (Destro *et al.*, 2017 ▸), possibly illustrating an effect inherent to experimental data.

In the case of calcium/magnesium cations, the literature values are noticeably smaller. However, this is not surprising since the Mg cation is expected to show a smaller volume than the Ca cation.

**Figure 3:**
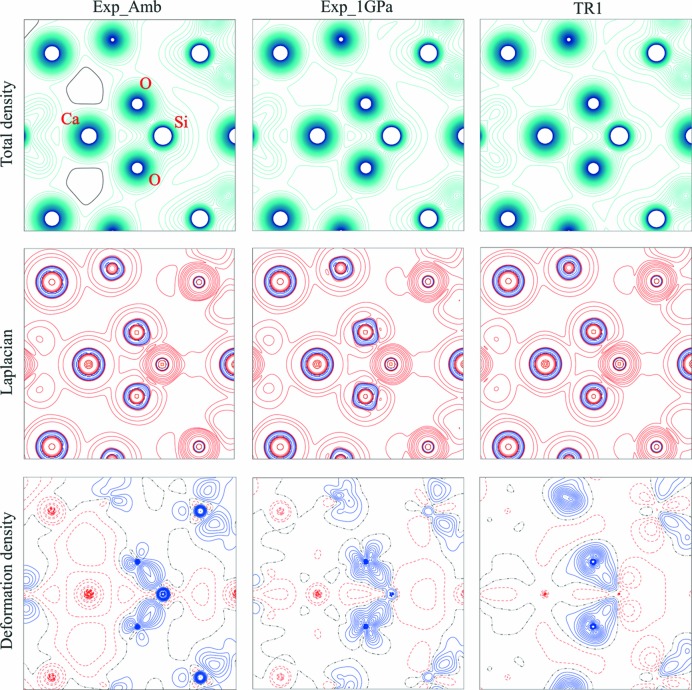
Maps of electron density distribution for the Ca–Si–O plane. Contour values for total density and deformation density are 0.1 e Å^−3^ and 0.05 e Å^−3^, respectively. For Laplacian maps, particular contours are ±2, 4, 8, 20, 40, 80, 200, 400 and 1000 e Å^−5^. Blue contours denote positive values and red contours correspond to negative values. The same color scheme is adopted for the deformation density maps.

Another way to identify certain differences between results obtained for Exp_Amb and Exp_1GPa is to compare contour maps for properties such as the total electron density, deformation electron density or Laplacian of the electron density. Such maps are presented in Fig. 3 ▸ for the Ca–Si–O plane.

In the total density plots (upper rows of Fig. 3 ▸), one can clearly see the aspherical shape of the valence electron density for all ions. However, the effect of the applied pressure is hardly seen even on the Laplacian maps. The most significant difference possible that can be observed is between the deformation density map for TR1 and for both experiments. In the case of theoretical calculations, the electron density around oxygen ions is shaped differently (more smooth) than in the case of the experimental results.

**Figure 4:**
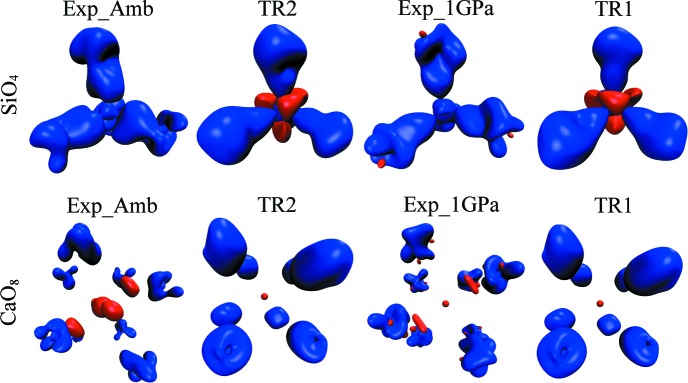
3D deformation electron density maps; (first row) SiO_4_, (second row) CaO_8_. From left to right: experimental ambient pressure Exp_Amb, theoretical calculations TR2, experimental high-pressure Exp_1GPa and corresponding theoretical calculations TR1. Blue contour: +0.1 e Å^−3^; red contour: −0.1 e Å^−3^.

All these different shapes of electron density on maps in Fig. 3 ▸ result from sections of 3D maps of electron density through particular planes defined by the Ca–O–Si plane. Corresponding 3D deformation electron density maps are displayed in Fig. 4 ▸ for SiO_4_ (first row) and CaO_8_ (second row), illustrating changes at the ±0.1 e Å^−3^ isosurfaces of the electron density.

As illustrated in Fig. 4 ▸, the theoretically calculated 3D shapes of deformation density are more similar to those obtained on the basis of experimental data in the case of SiO_4_ than in the case of CaO_8_. Si—O contacts have more covalent character than Ca—O contacts, which are more ionic. However, the resemblance between particular 3D deformation maps can be attributed to the type of data (theoretical results are similar to theoretical ones and experimental results are similar to the experimental ones) rather than the pressure value (or value of the *a* parameter).

All the above mentioned similarities between the electron density distribution at 1 GPa (synchrotron measurements) and at ambient pressure (laboratory measurements) as well as their consistency with the literature data describing isostructural compounds suggest two facts. Firstly, during the measurement at 1 GPa, enclosing the sample in a DAC did not prevent the acquisition of a dataset of quality good enough to refine a reasonable Hansen–Coppens multipole model of electron density. Secondly, the pressure difference between the two experiments considered was too small to observe significant differences in properties at the BCPs.

## 4. Conclusions   

We demonstrated that charge density analysis under pressure can be successful when several conditions are fulfilled. The main condition is to reach data completeness at the highest resolution using a sample environment (*i.e.* DAC) which is intrinsically very constraining (small-sized samples, shadowing effect of the DAC and diamond absorption of the incoming and outgoing X-ray beams). The joint use of a DAC with a large opening angle and high X-ray flux at a short wavelength (the shorter the better) available at synchrotrons is the first prerequisite. An adapted setup, such as the one available at the CRISTAL beamline at the SOLEIL synchrotron, giving the possibility of several degrees of freedom for the orientation of the sample is also mandatory to reach the completeness. In addition, owing to synchrotron radiation flux, the total time of such measurements is significantly shorter than the case of in-house data. In this work, the total time required for data collection for grossular at the CRISTAL beamline was less than 5 h. Within this time we obtained an excellent dataset, allowing us to refine the experimental electron density, which is comparable with the literature data and theoretical calculations.

The compound investigated crystallizes in the most complex cubic space group. However, by placing more than one piece of single crystal in the pressure chamber (in different orientations), it should be possible to successfully collect complete datasets for crystals with lower symmetry.

Such feasibility studies open new possibilities in mineralogy, particularly in studies of phase transitions and different mineralogical processes which from now on can be investigated at the level of detail of experimental electron densities. Such studies would be highly influential for geophysicists describing the way seismic waves pass through the Earth’s mantle and those working on mantle gravity models. Changing structures might also affect the way in which trace elements are incorporated into mantle minerals and, more importantly, how they are released during partial melting of the Earth’s mantle.

This study, performed on a real natural crystal, also demonstrates the type of quantitative experimental charge density studies that are possible for real minerals that potentially contain substitutions or are subject to variable compositions.

### 4.1. Data availability   

Data supporting the findings of this study are available from the correspondence author upon reasonable request. CCDC 1936438–1936441 entries contain the supplementary crystallographic data for grossular at ambient conditions and under pressure. These data can be obtained free of charge from the Cambridge Crystallographic Data Centre via https://www.ccdc.cam.ac.uk/data_request/cif.

## 5. Related literature   

The following references are cited in the supporting information: Abrahams & Geller (1958 ▸); Boettcher (1970 ▸); Conrad *et al.* (1999 ▸); Darco *et al.* (1996 ▸); Erba *et al.* (2014 ▸); Etschmann *et al.* (2001 ▸); Ganguly *et al.* (1993 ▸); Geiger & Armbruster (1997 ▸); Greaux *et al.* (2011 ▸, 2014 ▸); Hazen & Finger (1978 ▸); Henn & Meindl (2015 ▸); Lager *et al.* (1987 ▸); Meagher (1975 ▸); Meyer *et al.* (2010 ▸); Nobes *et al.* (2000 ▸); Oberti *et al.* (2006 ▸); Ottonello *et al.* (1996 ▸); Pavese *et al.* (2001 ▸); Prandl (1966 ▸); Rodehorst *et al.* (2002 ▸); Sawada (1997*a* ▸,*b* ▸,*c* ▸, 1999 ▸); Thirumalaisamy *et al.* (2016 ▸); Zhang *et al.* (1999 ▸).

## Supplementary Material

Crystal structure: contains datablock(s) Exp_1GPa, Exp_1GPa_multipole, Exp_Ambient, Exp_Ambient_multipole, TR1, TR2, TR3, TR4, TR5. DOI: 10.1107/S2052252520001955/fc5038sup1.cif

Supporting figures and tables. DOI: 10.1107/S2052252520001955/fc5038sup2.pdf

CCDC references: 1936439, 1936440, 1936441, 1973091

## Figures and Tables

**Table 1:** Selected crystal data for spherical and multipole refinements of grossular at ambient pressure and at 1 GPa pressure

Data source	Exp_Amb	Exp_1GPa
Spherical refinement		
Pressure (GPa)	Ambient	1
*a* (Å)	11.85877 (6)	11.87985 (9)
*V* (Å^3^)	1667.71 (3)	1676.61 (4)
*Z*, *F*(000)	8, 1792	8, 1792
*D*_*x*_ (Mg m^−3^)	3.588	3.569
Wavelength (Å)	0.7107	0.4166
μ (mm^−1^)	2.71	0.60
Crystal size (mm)	0.15 × 0.10 × 0.04	0.15 × 0.10 × 0.04
Absorption correction	Numerical absorption correction, *T*_min_ = 0.776, *T*_max_ = 0.918	Empirical multiscan, *T*_min_ = 0.61480, *T*_max_ = 1.00000
Measured reflections	40053	31213
Independent reflections	809	808
Observed reflections [*I* > 2σ(*I*)]	761	790
*R*_int_	0.033	0.061
θ values (°)	θ_max_ = 52.1, θ_min_ = 4.2	θ_max_ = 27.6, θ_min_ = 2.5
(sin θ/λ)_max_ (Å^−1^)	1.11	1.11
Range of *h*, *k*, *l*	*h* = −26→25, *k* = −26→22, *l* = −25→26	*h* = −24→16, *k* = −17→26, *l* = −14→21
Refinement on, parameters, reflections	*F* ^2^/17/809	*F* ^2^/17/808
*R*[*F* ^2^ > 2σ(*F* ^2^)], *wR*(*F* ^2^), *S*	0.016, 0.055, 1.21	0.035, 0.088, 1.42
Weighting scheme	*w* = 1/[σ^2^(*F*_o_^2^) + (0.0246*P*)^2^ + 0.6022*P*] where *P* = (*F*_o_^2^ + 2*F*_c_^2^)/3	*w* = 1/[σ^2^(*F*_o_^2^) + (0.025*P*)^2^ + 2.2749*P*] where *P* = (*F*_o_^2^ + 2*F*_c_^2^)/3
(Δ/σ)_max_	<0.001	<0.001
Δ〉_max_, Δ〉_min_ (e Å^−3^)	0.78, −0.70	0.50, −0.53
Diffractometer	Rigaku SuperNova four-circle diffractometer	Newport six-circle diffractometer
		
Multipole refinement		
Refinement on, parameters, reflections	*F* ^2^/43/725	*F* ^2^/43/737
*R*[*F* ^2^ > 2σ(*F* ^2^)], *R*(all)	0.015, 0.031	0.029, 0.034,
*wR*[*F* ^2^ > 2σ(*F* ^2^)], *wR*(all), *S*	0.019, 0.051, 1.578	0.030, 0.077, 2.39
Weighting scheme	*w* = 1/[σ^2^(*F*_o_^2^) + 0.02*P* ^2^ + 0.86*P*] where *P* = (*F*_o_^2^ + 2*F*_c_^2^)/3	*w* = 1/[σ^2^(*F*_o_^2^) + 2.65*P*] where *P* = (*F*_o_^2^ + 2*F*_c_^2^)/3
(Δ/σ)_max_	0	0
Δ〉_max_, Δ〉_min_ (e Å^−3^)	0.706, −0.498	0.741, −0.848

**Table 2:** Comparison of the unit-cell parameter *a* and interionic distances (Å) in the structures of grossular obtained experimentally (synchrotron and in-house diffractometer) and theoretically (TR*i*, *i* = 1–5) Computations without a set external pressure are marked with n/a.

	Exp_1GPa	TR1	Exp_Amb	TR2	TR3	TR4	TR5
*p* (GPa)	1.00 (1)	n/a	Ambient	n/a	n/a	1.0	10.0
*a*	11.8799 (1)	11.8799 (1)	11.8588 (1)	11.8588 (1)	11.8295 (1)	11.8080 (1)	11.6320 (1)
Ca—O′	2.3303 (4)	2.3276 (2)	2.3257 (2)	2.3263 (2)	2.3218 (2)	2.3184 (2)	2.2913 (2)
Ca—O′′	2.4912 (4)	2.4910 (2)	2.4892 (2)	2.4840 (2)	2.4745 (2)	2.4674 (2)	2.4099 (2)
Si—O	1.6513 (4)	1.6595 (2)	1.6466 (2)	1.6579 (2)	1.6555 (2)	1.6539 (2)	1.6397 (2)
Al—O	1.9319 (4)	1.9236 (2)	1.9288 (2)	1.9194 (2)	1.9149 (2)	1.9115 (2)	1.8843 (2)

**Table:** 

	Exp_1GPa					
ADPs	*U*_11_	*U*_22_	*U*_33_	*U*_12_	*U*_13_	*U*_23_
Ca	0.00595 (7)	0.00595 (7)	0.00381 (9)	0.00086 (5)	0	0
Si	0.00358 (9)	0.00358 (9)	0.00333 (12)	0	0	0
Al	0.0026 (1)	0.0026 (1)	0.0026 (1)	−0.00012 (6)	−0.00012 (6)	−0.00012 (6)
O	0.00448 (14)	0.00596 (14)	0.00505 (14)	0.00043 (10)	0.00062 (10)	−0.00032 (10)

**Table:** 

	Exp_Amb					
ADPs	*U*_11_	*U*_22_	*U*_33_	*U*_12_	*U*_13_	*U*_23_
Ca	0.00588 (4)	0.00588 (4)	0.00357 (5)	0.00090 (2)	0	0
Si	0.00343 (5)	0.00343 (5)	0.00314 (7)	0	0	0
Al	0.00273 (5)	0.00273 (5)	0.00273 (5)	−0.00010 (3)	−0.00010 (3)	−0.00010 (3)
O	0.00444 (7)	0.00608 (7)	0.00517 (7)	0.00034 (5)	0.00071 (5)	−0.00035 (5)

**Table 4:** Properties of the charge density ρ(*r*_c_) (e Å^−3^) and the Laplacian ∇^2^ρ(*r*_c_) (e Å^−5^) at the (3, −1) BCPs of grossular *d*_1-bcp_ and *d*_2-bcp_ (Å) denote the distances from the BCP to atoms 1 and 2, respectively. In the case of theoretical refinements (TR*i*), the left and right values correspond to results from *XD2016* and *TOPOND14*, respectively.

*X*—*Y* interaction		Si—O	Al—O	Ca—O^I^ †	Ca—O^II^ †
*d*_1-bcp_	Exp_Amb	0.694	0.833	1.194	1.273
	Exp_1GPa	0.696	0.853	1.191	1.266
	TR1	0.705 / 0.685	0.829 / 0.804	1.181 / 1.163	1.257 / 1.234
	TR2	0.704 / 0.685	0.827 / 0.803	1.180 / 1.162	1.254 / 1.231
	TR3	0.703 / 0.684	0.826 / 0.801	1.178 / 1.160	1.249 / 1.228
	TR4	0.703 / 0.684	0.824 / 0.800	1.176 / 1.159	1.246 / 1.225
	TR5	0.698 / 0.679	0.815 / 0.792	1.165 / 1.149	1.220 / 1.203
	Pyrope[Table-fn tfn1]	0.692	0.798	0.969	1.039
*d*_2-bcp_	Exp_Amb	0.953	1.102	1.132	1.244
	Exp_1GPa	0.957	1.089	1.143	1.231
	TR1	0.955 / 0.974	1.095 / 1.120	1.151 / 1.165	1.237 / 1.257
	TR2	0.954 / 0.973	1.093 / 1.117	1.150 / 1.164	1.233 / 1.253
	TR3	0.953 / 0.971	1.090 / 1.114	1.148 / 1.162	1.228 / 1.247
	TR4	0.951 / 0.970	1.088 / 1.112	1.147 / 1.159	1.224 / 1.243
	TR5	0.942 / 0.960	1.070 / 1.093	1.131 / 1.143	1.191 / 1.206
	Pyrope[Table-fn tfn1]	0.943	1.086	1.228	1.294
ρ(*r*_c_)	Exp_Amb	1.15	0.51	0.19	0.18
	Exp_1GPa	1.06	0.53	0.25	0.19
	TR1	1.07 / 0.90	0.40 / 0.41	0.29 / 0.28	0.16 / 0.18
	TR2	1.07 / 0.91	0.40 / 0.41	0.30 / 0.28	0.16 / 0.18
	TR3	1.08 / 0.91	0.41 / 0.41	0.30 / 0.28	0.16 / 0.18
	TR4	1.09 / 0.92	0.42 / 0.42	0.30 / 0.28	0.17 / 0.18
	TR5	1.12 / 0.95	0.44 / 0.45	0.32 / 0.30	0.19 / 0.21
	Pyrope[Table-fn tfn1]	0.89	0.49	0.27	0.21
∇^2^ρ(*r*_c_)	Exp_Amb	8.5	3.3	5.1	2.7
	Exp_1GPa	9.2	1.0	4.4	2.8
	TR1	6.0 / 17.2	5.0 / 8.0	4.2 / 5.1	3.3 / 3.4
	TR2	6.0 / 17.4	5.0 / 8.2	4.2 / 5.1	3.4 / 3.5
	TR3	6.0 / 17.6	5.0 / 8.3	4.2 / 5.2	3.4 / 3.6
	TR4	6.0 / 17.7	5.1 / 8.4	4.3 / 5.2	3.5 / 3.6
	TR5	6.8 / 19.0	6.0 / 9.3	4.7 / 5.6	4.1 / 4.2
	Pyrope[Table-fn tfn1]	17.0	8.3	3.1	1.8

† Destro *et al.* (2017 ▸) (Mg instead of Ca).

‡ Two non-equivalent Ca—O contacts exist in this structure.

**Table 5:** Integrated volume of atomic basin *V* (Å^3^) and net charge *Q* (e) for the four atomic species of grossular

	Ca		Si		Al		O	
	*V*	*Q*	*V*	*Q*	*V*	*Q*	*V*	*Q*
Exp_Amb	11.28	1.96	5.63	2.24	4.78	1.95	12.32	−1.38
Exp_1GPa	11.22	1.66	4.49	2.79	3.87	2.21	12.87	−1.47
TR1	11.13	1.65	3.41	3.05	3.25	2.67	13.28	−1.60
TR2	11.09	1.65	3.39	3.05	3.23	2.67	13.21	−1.60
TR3	11.01	1.65	3.32	3.07	3.20	2.67	13.12	−1.60
TR4	10.95	1.64	3.30	3.07	3.18	2.67	13.05	−1.60
TR5	10.50	1.63	3.18	3.08	3.09	2.67	12.45	−1.60
Pyrope†	6.98	1.62	5.74	2.82	4.16	2.69	11.72	−1.55

† Destro *et al.* (2017 ▸).

## References

[bb100] Abrahams, S. & Geller, S. (1958). *Acta Cryst.* **11**, 437–441.

[bb1] Antao, S. M. (2013). *Phys. Chem. Miner.* **40**, 705–716.

[bb2] Bianchi, R., Forni, A., Cámara, F., Oberti, R. & Ohashi, H. (2005*a*). *Phys. Chem. Miner.* **34**, 519–527.

[bb3] Bianchi, R., Forni, A. & Oberti, R. (2005*b*). *Phys. Chem. Miner.* **32**, 638–645.

[bb4] Blessing, R. H. (1995). *Acta Cryst.* A**51**, 33–38. 10.1107/s0108767394005726 7702794

[bb101] Boettcher, A. L. (1970). *J. Petrol.* **11**, 337–379.

[bb5] Casati, N., Genoni, A., Meyer, B., Krawczuk, A. & Macchi, P. (2017). *Acta Cryst.* B**73**, 584–597. 10.1107/S2052520617008356 28762969

[bb6] Casati, N., Kleppe, A., Jephcoat, A. P. & Macchi, P. (2016). *Nat. Commun.* **7**, 10901. 10.1038/ncomms10901 PMC4799374 26979750

[bb102] Conrad, P. G., Zha, C. S., Mao, H. K. & Hemley, R. J. (1999). *Am. Mineral.* **84**, 374–383.

[bb103] Darco, P., Fava, F. F., Dovesi, R. & Saunders, V. R. (1996). *J. Phys. Condens. Matter*, **8**, 8815–8828.

[bb8] Destro, R., Ruffo, R., Roversi, P., Soave, R., Loconte, L. & Lo Presti, L. (2017). *Acta Cryst.* B**73**, 722–736. 10.1107/S2052520617006102 PMC6181205 28762982

[bb9] Dolomanov, O. V., Bourhis, L. J., Gildea, R. J., Howard, J. A. K. & Puschmann, H. (2009). *J. Appl. Cryst.* **42**, 339–341.

[bb10] Dovesi, R., Erba, A., Orlando, R., Zicovich-Wilson, C. M., Civalleri, B., Maschio, L., Rérat, M., Casassa, S., Baima, J., Salustro, S. & Kirtman, B. (2018). *WIREs Comput. Mol. Sci.* **8**, e1360.

[bb11] Dovesi, R., Saunders, V. R., Roetti, C., Orlando, R., Zicovich-Wilson, C. M., Pascale, F., Civalleri, B., Doll, K., Harrison, N. M., Bush, I. J., D’Arco, P., Llunell, M., Causà, M., Noël, Y., Maschio, L., Erba, A., Rerat, M. & Casassa, S. (2017). *CRYSTAL17 User’s Manual*. University of Torino, Italy.

[bb12] Downs, R. T. (2003). *Am. Mineral.* **88**, 556–566.

[bb13] Erba, A., Ferrabone, M., Orlando, R. & Dovesi, R. (2013). *J. Comput. Chem.* **34**, 346–354. 10.1002/jcc.23138 23081746

[bb104] Erba, A., Mahmoud, A., Belmonte, D. & Dovesi, R. (2014). *J. Chem. Phys.* **140**, 124703. 10.1063/1.4869144 24697466

[bb105] Etschmann, B., Streltsov, V., Ishizawa, N. & Maslen, E. N. (2001). *Acta Cryst.* B**57**, 136–141. 10.1107/s0108768100019923 11262427

[bb14] Fabbiani, F. P. A., Dittrich, B., Pulham, C. R. & Warren, J. E. (2011). *Acta Cryst.* A**67**, C376.

[bb15] Farrugia, L. J. (2012). *J. Appl. Cryst.* **45**, 849–854.

[bb106] Ganguly, J., Cheng, W. & Oneill, H. (1993). *Am. Mineral.* **78**, 583–593.

[bb17] Gatti, C. & Casassa, S. (2013). *TOPOND14 User’s Manual*, CNR-ISTM of Milano, Italy.

[bb16] Gatti, C., Saunders, V. R. & Roetti, C. (1994). *J. Chem. Phys.* **101**, 10686–10696.

[bb107] Geiger, C. A. & Armbruster, T. (1997). *Am. Mineral.* **82**, 740–747.

[bb18] Gibbs, G. V., Cox, D. F., Rosso, K. M., Kirfel, A., Lippmann, T., Blaha, P. & Schwarz, K. (2005). *Phys. Chem. Miner.* **32**, 114–125.

[bb19] Gibbs, G. V., Ross, N. L., Cox, D. F. & Rosso, K. M. (2014). *Am. Mineral.* **99**, 1071–1084.

[bb109] Greaux, S., Andrault, D., Gautron, L., Bolfan N. & Mezouar, M. (2014). *Phys. Chem. Miner.* **41**, 419–429.

[bb108] Greaux, S., Kono, Y., Nishiyama, N., Kunimoto, T., Wada, K. & Irifune, T. (2011). *Phys. Chem. Miner.* **38**, 85–94.

[bb20] Hansen, N. K. & Coppens, P. (1978). *Acta Cryst.* A**34**, 909–921.

[bb110] Hazen, R. M. & Finger, L. W. (1978). *Am. Mineral.* **63**, 297–303.

[bb111] Henn, J. & Meindl, K. (2015). *Mater. Chem. Phys.* **1**, 417–430.

[bb21] Ivanov, Y. V. & Belokoneva, E. L. (2007). *Acta Cryst.* B**63**, 49–55. 10.1107/S0108768106041681 17235193

[bb22] Ivanov, Yu. V., Belokoneva, E. L., Protas, J., Hansen, N. K. & Tsirelson, V. G. (1998). *Acta Cryst.* B**54**, 774–781.

[bb23] Kamiński, R., Domagała, S., Jarzembska, K. N., Hoser, A. A., Sanjuan-Szklarz, W. F., Gutmann, M. J., Makal, A., Malińska, M., Bąk, J. M. & Woźniak, K. (2014). *Acta Cryst.* A**70**, 72–91. 10.1107/S2053273313028313 24419172

[bb24] Kirfel, A. & Gibbs, G. V. (2000). *Phys. Chem. Miner.* **27**, 270–284.

[bb25] Kuntzinger, S. & Ghermani, N. E. (1999). *Acta Cryst.* B**55**, 273–284. 10.1107/s0108768198013536 10927368

[bb112] Lager, G., Rossman, G., Rotella, F. & Schultz, A. (1987). *Am. Mineral.* **72**, 766–768.

[bb26] Li, R., Liu, J., Bai, L., Tse, J. S. & Shen, G. (2015). *Appl. Phys. Lett.* **107**, 072109.

[bb27] Luaña, V., Costales, A., Mori-Sánchez, P. & Pendás, A. M. (2003). *J. Phys. Chem. B*, **107**, 4912–4921.

[bb113] Meagher, E. (1975). *Am. Mineral.* **60**, 218–228.

[bb114] Meyer, A., Pascale, F., Zicovich-Wilson, C. M. & Dovesi, R. (2010). *Int. J. Quantum Chem.* **110**, 338–351.

[bb115] Nobes, R. H., Akhmatskaya, E. V., Milman, V., Winkler, B. & Pickard, C. J. (2000). *Comput. Mater. Sci.* **17**, 141–145.

[bb116] Oberti, R., Quartieri, S., Dalconi, M.C., Boscherini, F., Iezzi, G., Boiocchi, M. & Eeckhout, S.G. (2006). *Am. Mineral.* **91**, 1230–1239.

[bb117] Ottonello, G., Bokreta, M. & Sciuto, P. F. (1996). *Am. Mineral.* **81**, 429–447.

[bb118] Pavese, A., Diella, V., Pischedda, V., Merli, M., Bocchio, R. & Mezouar, M. (2001). *Phys. Chem. Miner.* **28**, 242–248.

[bb119] Prandl, W. (1966). *Z. Kristallogr.* **123**, 81–116.

[bb28] Prencipe, M., Tribaudino, M. & Nestola, F. (2003). *Phys. Chem. Miner.* **30**, 606–614.

[bb7] Rigaku Oxford Diffraction (2015). *CrysAlis PRO*. Rigaku Corporation, Tokyo, Japan.

[bb120] Rodehorst, U., Geiger, C. A. & Armbruster, T. (2002). *Am. Mineral.* **87**, 542–549.

[bb121] Sawada, H. (1997*a*). *J. Solid State Chem.* **132**, 300–307.

[bb122] Sawada, H. (1997*b*). *J. Solid State Chem.* **132**, 432–433.

[bb123] Sawada, H. (1997*c*). *J. Solid State Chem.* **134**, 182–186.

[bb124] Sawada, H. (1999). *J. Solid State Chem.* **142**, 273–278.

[bb29] Sheldrick, G. M. (2008). *Acta Cryst.* A**64**, 112–122. 10.1107/S0108767307043930 18156677

[bb30] Sheldrick, G. M. (2015). *Acta Cryst.* C**71**, 3–8.

[bb125] Thirumalaisamy, T. K., Saravanakumar, S., Butkute, S., Kareiva, A. & Saravanam, R. (2016). *J. Mater. Sci. Mater. Electron.* **27**, 1920–1928.

[bb31] Volkov, A., Macchi, P., Farrugia, L., Gatti, C., Mallinson, P., Richter, T. & Koritsanszky, T. (2016). *XD2016*. University at Buffalo, State University of New York, NY, USA; University of Milan, Italy; University of Glasgow, UK; CNRISTM, Milan, Italy; Middle Tennessee State University, TN, USA; and Freie Universität, Berlin, Germany.

[bb32] Yamanaka, T., Okada, T. & Nakamoto, Y. (2009). *Phys. Rev. B*, **80**, 094108.

[bb126] Zhang, L., Ahsbahs, H., Kutoglu, A. & Geiger, C. A. (1999). *Phys. Chem. Miner.* **27**, 52–58.

