# Supplementary material for: Experimental charge density of grossular under pressure – a feasibility study
Source: IUCrJ. 2020 Mar 7;7(Pt 3):383–92. doi: 10.1107/S2052252520001955 (PMC7201277; doi:10.1107/S2052252520001955)
Supplement: Supplementary file 2 [file m-07-00383-sup2.pdf]

IUCrJ

Volume 7 (2020)

Supporting information for article:

Experimental charge density of grossular under pressure – a feasibility study

Roman Gajda, Marcin Stachowicz, Anna Makal, Szymon Sutuła, Jan Parafiniuk, Pierre Fertey and Krzysztof Woźniak

Experimental Charge Density of Grossular Under Pressure - a Feasibility Study

Roman Gajda[a], Marcin Stachowicz[a,b], Anna Makal[a], Szymon Sutuła[a], Jan Parafiniuk[b], Pierre Fertey[c], Krzysztof Woźniak[a]*

[a]Biological and Chemical Research Centre, Department of Chemistry, University of Warsaw, Żwirki i Wigury 101, 02-093 Warszawa, Poland.
[b]Institute of Geochemistry, Mineralogy and Petrology, Department of Geology, University of Warsaw, Żwirki i Wigury 93, 02-089 Warszawa, Poland
[c]Synchrotron SOLEIL, L'Orme des Merisiers - Saint Aubin, B.P. 48, 91 192 Gif-sur-Yvette Cedex, France

***Corresponding author:** kwozniak@chem.uw.edu.pl

Keywords: Grossular, Charge Density, Pressure, synchrotron, Multipole refinement

Contents

1. Additional information on garnets……………………………………………………………..3

2. Scanning electron microscope………………………………………………………………………4

Figure 1S. A scanning electron microscope energy-dispersive spectrum………………………………..4

3. The unit cell parameter *a*……………………………………………………………………………5

Table 1S. Values of the unit cell parameter *a* obtained at ambient pressure……………………………..5

Table 2S. Values of the unit cell parameter *a* obtained under pressure…………………………………….6

Figure 2S. The unit cell parameter *a* of grossular as a function of temperature…………………………7

Figure 3S. Scatter of the unit cell parameter *a* of grossular measured at room temperature……………8

Table 3S. Comparison of interatomic distances in structures of pure grossular…………………………8

Figure 4S. Unit cell parameter *a* of grossular in function of pressure………………………………….10

4. Experimental deformation map……………………………………………………………………11

Figure 5S. Experimental deformation map (with multipolar phases). Plane Ca-Si-O…………………11

5. Properties of the charge density at the (3,-1) BCPs of grossular……………………………………….12

Table 4S. Properties of the charge density at the (3,-1) BCPs of grossular…………………………….12

6. High pressure, high resolution, single crystal X-ray diffraction data collection strategy………………14

Table 5S. Definition of the high-pressure data collection strategy……………………………………..14

7. Quality of hkl data sets and the fitted models…………………………………………………………..15

7.1. Quality of fitted models………………………………………………………………………………15

Figure 6S. Ratios of the sums of observed and calculated structure-factor amplitudes as a function of sin θ/λ……………………………………………………………………………………………………15

Figure 7S. Fractal dimension (d^f) vs. residual density (ρ_0)…………………………………………….16

Figure 8S. F observed versus F calculated……………………………………………………………..17

Figure 9S. Normal probability plots…………………………………………………………………18

Figure 10S. Normal distribution of residual electron density……………………………………………..19

Figure 11S. Residual electron density maps……………………………………………………………20

Figure 12S. Comparison of theoretical calculations for different pressures……………………………20

Figure 13S. 3D deformation electron maps of SiO4……………………………………………………21

Figure 14S. 3D deformation electron density maps of CaO8……………………………………………..21

8. Steps of the (multipolar) refinement………………………………………………………………23

9. Theoretical results………………………………………………………………………………24

Table 6S. ADPs for atoms in structure of grossular obtained theoretically…………………………….24

10. References………………………………………………………………………………………..25

1. Additional information on garnets.

Garnets belong to nesosilicate (orthosilicate) minerals, important and common in nature. They are usually divided into two series: pyralspite [almandine $Fe_3Al_2(SiO_4)_3$, pyrope $Mg_3Al_2(SiO_4)_3$, spessartine $Mn_3Al_2(SiO_4)_3$] and ugrandite [andradite $Ca_3Fe_2(SiO_4)_3$, grossular $Ca_3Al_2(SiO_4)_3$ and uvarovite $Ca_3Cr_2(SiO_4)_3$].

Grossular forms continuous solid solutions with uvarovite $Ca_3Cr_2(SiO_4)_3$ and andradite $Ca_3Fe_2(SiO_4)_3$**.** In grossular structure, calcium may also be partially replaced by Fe^{2+}, Mn^{2+}, Mg^{2+}, sometimes in smaller amounts even by Sn^{2+} and Zn^{2+}. Beside Cr^{3+} and Fe^{3+}, aluminum may be partly replaced by Mn^{3+} and V^{3+}. A vast diversity of chemical composition found in nature is the reason for distinguishing numerous varieties of grossular with their own names, used especially in gemmology *e.g.* orange hessonite (Fe content), green tsavorite (V) or massive transvaal jade (Cr). In grossular variety known as hibschite, four hydroxide ions substitute part of the orthosilicate ion. When 4OH groups prevail in the structure over orthosilicate, a new mineral – katoite is distinguished. The extensive solid substitution series grossular – katoite is often informally called hydrogrossular or hydrogarnet.

Grossular frequently forms isometric, euhedral crystals in forms of dodecahedra or trapezohedra and their combinations. Faces of rare hexoctahedra are also sometimes observed. Mineral occurs as granular or compact masses as well. Grossular is a typical component of metamorphic rocks. It commonly forms in contact metamorphic condition as a constituent of skarns or calc-silicate hornfelses. This garnet occurs also as a product of regionally metamorphosed impure limestones or marls. It is one of the main constituents of rocks which have undergone calcium metasomatism like rodingites associated with serpentinite bodies.

Experimental work on the synthesis and stability of grossular summarized *e.g.* by Boettcher (1970) showed that this garnet is stable at atmospheric pressure to approximately 850°C. Investigations of garnet structures focused on electron density were conducted since the nineties of the previous century. Some of them described theoretical approach and results of *ab initio* CRYSTAL calculations (D'Arco *et al.*, 1996; Meyer *et al.*, 2010). Attempts at synchrotron studies were also made (Etschmann *et al.*, 2001). In some cases maximum entropy methods (MEM) were also used to obtain electron density distribution (Thirumalaisamy *et al.*, 2016). Systematic investigations of many garnet-type structures were also performed just by single-crystal X-ray measurements (Sawada, 1997a; Sawada, 1997b; Sawada, 1997c; Sawada, 1999).

2. Scanning electron microscopy.

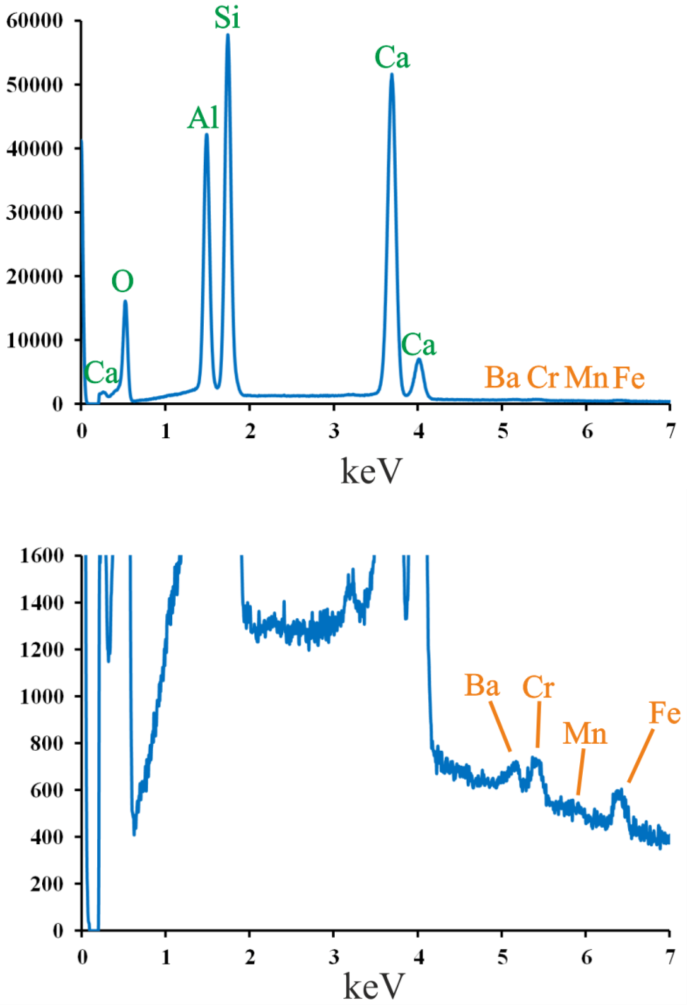

Figure 1S. A scanning electron microscope energy-dispersive spectrum.
A general view and zoom on area where peaks from admixtures are visible.

Analysis of different grains of grossular showed that except calcium, aluminium, silicon and oxygen, there are also traces of iron, barium, chromium and manganese. The measurement showed that most significant substitution in this specimen was substitution of iron, the concentration of which varied between 0.26% to 1.82%.

3. The unit cell parameter *a*.

The unit cell parameter ***a*** (**Table 1**) which we obtained under pressure (sample **Exp_1GPa**) was surprisingly larger than the one at the ambient conditions (**Exp_Amb**) (11.87985(9)Å and 11.85877(6)Å, respectively). One would expect a decrease of the ***a*** parameter when pressure is applied, as already been reported in several studies (Greaux *et al.*, 2011; Greaux *et al.*, 2014; Hazen &Finger, 1978; Pavese *et al.*, 2001)). The increase observed here is *ca.* 0.021Å, which is *ca.* 0.18% of the ambient unit cell constant. Sample-to-sample standard deviation on the unit cell parameters deduced from multiple measurements of oxalic acid single crystals cell parameters (Kaminski *et al.*, 2014) can serve as reference values of the sample standard deviation (σ_{ssd}) on the ***a*** parameters determined in this work. Though the observed difference between the 1GPa and ambient pressure values is small, it is however significantly larger (>5 σ_{ssd}) than the reference sample standard deviation deduced from the oxalic acid single crystals (i.e. σ_{ssd}~0.004 Å).This unexpected relation between the values of the unit cell parameter at ambient conditions and under 1GPa pressure may result from the fact that we performed only one measurements at both conditions (**Exp_Amb** and **Exp_1GPa**). Note also that the values determined at the two different pressure values were obtained from datasets measured with two different instruments. In order to clarify the origin of this difference, we performed subsequent multiple measurements at ambient pressure and at **1 GPa** using our *in-house* diffractometer.

Firstly, we have collected data for ten different single crystals of grossular at ambient pressure, just to determine the unit cell parameter on the basis of several hundred reflections (see Table 1S). X-ray structural measurements were conducted on Agilent Technologies SuperNova Dual Source with the MoKα radiation (λ = 0.71073 Å). The values of ***a*** varied between 11.8456Å and 11.8819Å with the average value equal to 11.8575Å. The calculated sample standard deviation was 0.0104Å.

Table 1S. Values of the unit cell parameter ***a*** obtained at ambient pressure.

**a [Å]**	**Resolution sinθ/λ [Å^{-1}]**
11.8819(6)	0.68
11.8572(3)	0.68
11.8607(2)	0.68
11.8468(4)	0.68
11.8644(3)	0.68
11.85067(13)	0.68
11.8544(3)	0.68
11.8456(2)	0.68
11.8546(2)	0.68
11.85851(16)	0.68
Ave	11.8575
ssd	0.0104

The same procedure was repeated for measurements under pressure close to 1 GPa on 11 different grossular crystals. The cell parameter was determined using the same strategy as the one at ambient pressure. After each fast data collection, the final pressure was determined.

For the ambient pressure and 1GPa short data sets, the lattice parameters were obtained by least-squares fits as implemented in the CrysAlis CCD software suite. Data were reduced using the CrysAlis RED program taking into account the proper opening angle of the DAC and the resolution limit corresponding to each measurement. The obtained values of the unit cell parameter **a** are summarized in **Table 2S**. At about 1GPa, the values of *a* varied from 11.7903Å up to 11.8990Å with the average value equal to 11.8506Å. Calculated sample standard deviation was significantly larger, 0.035Å.

Table 2S. Values of the unit cell parameter ***a*** obtained under pressure.

**Pressure [GPa]**	**a [Å]**	**Resolution sinθ/λ [Å^{-1}]**
**1.02**	11.874(3)	0.57
**1.10**	11.833(3)	0.56
**0.89**	11.899(4)	0.56
**1.07**	11.8560(9)	0.65
**1.04**	11.8391(13)	0.76
**0.98**	11.8578(11)	0.57
**1.10**	11.7901(8)	0.72
**0.92**	11.8729(11)	0.54
**1.04**	11.7903(7)	0.71
**0.99**	11.8864(8)	0.56
**1.02**	11.8539(7)	0.75
**Ave a**	11.8502	
**ssd**	0.035	

We also decided to check how much the value of the ***a*** parameter of grossular varied in studies published so far. There are 54 records regarding the structure of grossular in the Inorganic Crystal Structure Database (ICSD). However, 25 of them describe structures with significant substitutions of other elements such as iron, manganese, magnesium or titanium in the structure of grossular. There is also one case, where atomic positions of calcium, aluminum and silicon are partially substituted by scandium (Oberti *et al.*, 2006). The rest of the records (29 out of 54) are defined as pure structures of grossular consisting only of Ca, Al, Si and O atoms/ions. Because concentration of iron in our sample was smaller than 2%, we would rather compare our results with literature data describing grossular structures without any significant substitution.

First, let us discuss the a parameter of the high-resolution dataset collected at ambient pressure (11.85877(6) Å). This particular dataset was collected at room temperature. The unit cell parameter value can be compared to values from published studies conducted at different temperatures (Geiger & Armbruster, 1997; Meagher, 1975; Rodehorst *et al.*, 2002). A compilation of the varations of the ***a*** parameter as a function of the temperature is displayed on Figure 2S. The different studies seem consistent and one clearly sees that the difference observed in our work (red dot on Figure 2S, deviation of ca. 0.01 Å) can't be explain by a temperature effect, which would imply a temperature 100°C higher than expected. Note that there are also some other single results measured below 100K (Lager et al., 1987; Prandl, 1966), which differs significantly from the general trend, with a deviation amplitude in the same range as our result. Note also that the parameter ***a*** value determined in this work was obtained from a high resolution data set whereas the literature parameters ***a*** came from routine experiments which may be a source of significant discrepancies.

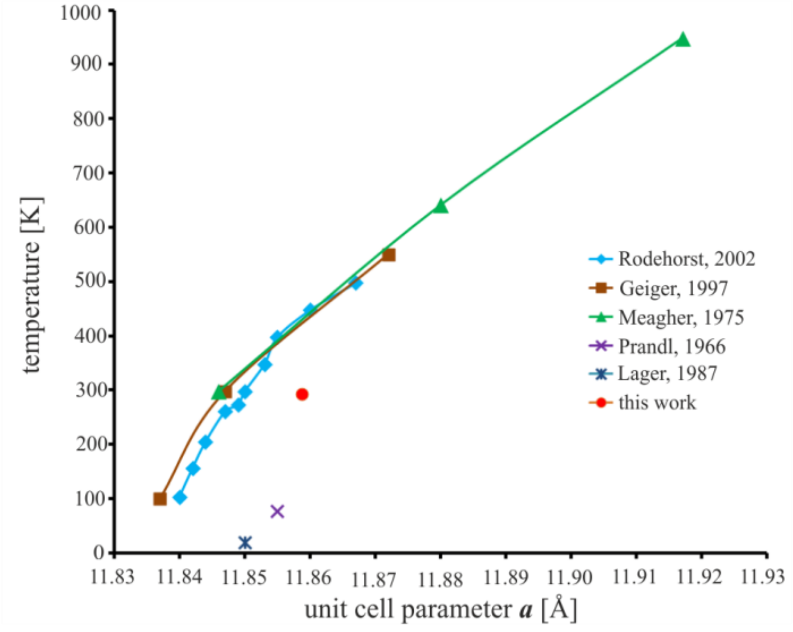

Figure 2S. The unit cell parameter ***a*** of grossular as a function of temperature.

Let us now compare our parameter ***a*** values with the literature data collected at the same temperature. Many papers present structural data of grossular collected at room temperature (293 K) or close to room temperature (298 K) (Sawada, 1999; Geiger & Armbruster, 1997; Meagher, 1997; Prandl, 1966; Rodehorst *et al.*, 2002; Abrahams & Geller, 1958; Ganguly *et al.*, 1993; Hazen & Finger, 1978, Ottonello *et al.*, 1996). Data from these studies are gathered in Fig. 3S showing how result for pure grossular at room temperature are scattered. Single red dot represents the parameter ***a*** determined in this work (high-resolution measurement) at ambient pressure.

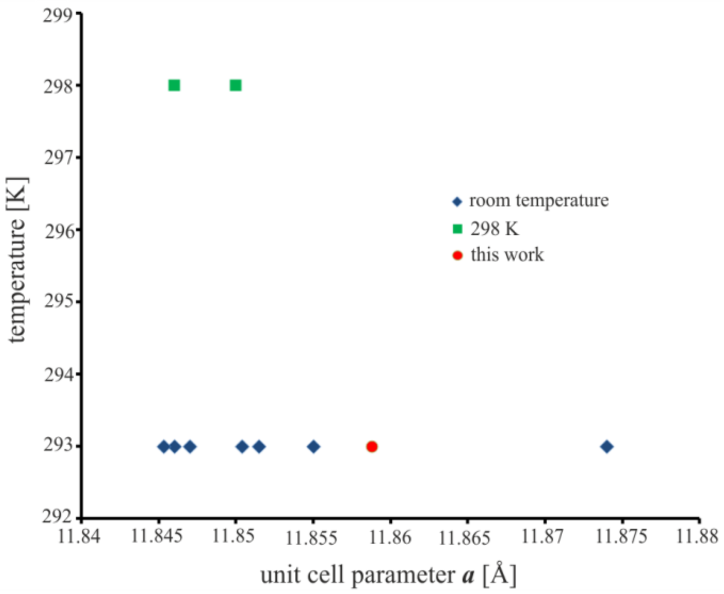

Figure 3S. Scatter of the unit cell parameter ***a*** of grossular measured at room temperature and ambient pressure.

Table 3S. Comparison of interatomic distances in structures of pure grossular.

a [Å]	Ca - O [Å]	Al - O [Å]	Si - O [Å]	References
11.874(4)	2.49(2)<br>2.33(2)	1.95(2)	1.64(2)	Abrahams & Geller, 1958
11.846(2)	2.492(5)<br>2.319(5)	1.921(5)	1.647(5)	Meagher, 1975
11.855(14)	2.484<br>2.328	1.926	1.649	Prandl, 1966
11.8504(4)	2.4873(9)<br>2.3223(9)	1.9269(9)	1.6466(9)	Sawada, 1999
11.847(1)	2.487(1)<br>2.322(1)	1.926(1)	1.646(1)	Geiger & Armbruster, 1997
11.850(1)	2.487<br>2.323	1.928	1.645	Rodehorst et al., 2002
11.846(1)	2.490<br>2.316	1.929	1.643	Sawada, 1997b
11.8515(2)	2.485<br>2.318	1.924	1.652	Ganguly et al. 1993
11.8453	2.504<br>2.325	1.926	1.633	Ottonello et al. 1996
11.85877 (6)	2.3256(3)<br>2.4889(3)	1.9291(2)	1.6465(3)	This work

There is only one literature ***a*** value which is larger than our result. However, the values of ***a*** are scattered between 11.846 Å and 11.874 Å. A sample standard deviation (ssd) for the unit cell parameter ***a*** calculated on the basis of these values is equal to 0.009 Å. Taking into account this ssd value, our value of the parameter ***a*** is still reasonable. Furthermore, we know that the grossular crystal used in this study is not absolutely pure. It contains no more than 2% of iron. This iron most probably substitutes the aluminum cations. It could also be one of the reason that our parameter ***a*** is slightly larger than most of literature data. As it was already described in details by Antao (2013),

the Ca-O, Al-O and Si-O distances and the ***a*** parameter are not independent one of another. As one distance changes because of substitution, it drags the other distances along by a minor amount. For example, if substitutions occur at the Al site, Al-O, and the unit cell parameter ***a,*** vary significantly, then also the Ca-O and Si-O distances change by a small amount. As seen from Table 2S, the Al-O distance is equal to 1.9291(2) Å and is one of the longest. So one may say that this slightly longer parameter **a** and interatomic distances are likely to be changed due to substitution of the Al by Fe. However, other literature data (Hazen & Finger, 1978) shows a similar Al-O distance equal 1.929 Å, and in this case, the ***a*** parameter is one of the shortest (11.846(1)Å). In fact since O is the only atom with refinable positions; increase in Al/Fe – O might theoretically be compensated by decrease in Si – O, independently of the a value. Note however that due to the Vegard low, the a dependency to the iron substitution rate is likely to happen.

At last, we have also compared how the unit cell parameter ***a*** changes as the function of applied pressure. The volume-pressure relations for grossular have been widely investigated by many authors over the last forty years. Most recently, Gréaux and co-workers (2011) conducted experiments to establish thermoelastic parameters for synthetic grossular and Erba and co-workers (2014) calculated them with the use of *ab initio* computations. In Fig. S8, we present the literature values of the unit cell parameter ***a*** as a function of pressure (Hazen & Finger, 1978; Pavese et al., 2011; Zhang et al., 1999; Greaux et al., 2011). On this figure, the single red dot denotes the value determined form the data collected at 1GPa (synchrotron measurements). The green squares illustrate our in-house measurements under pressure. As displayed in Fig. S8, our structural measurements under pressure using the in-house diffractometer correspond very well with those already published, especially with work of Pavese et al. (2001). That is why the deviation of the parameter ***a*** for the synchrotron dataset at 1 GPa could be likely related to the specific calibration of the instrument used at the CRISTAL beamline at SOLEIL and to the inherent misalignment of the sample located in the DAC with respect to the diffractometer center along the beam direction, as explained in & 2.1.3 of the manuscript..

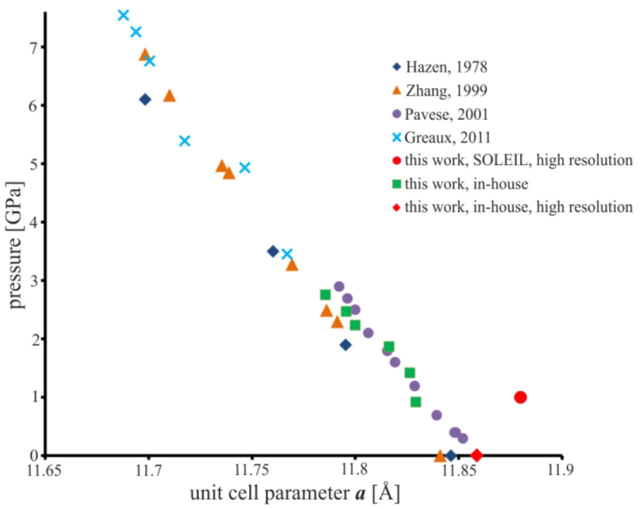

Figure 4S. Unit cell parameter ***a*** of grossular in function of pressure.

While the value of ***a*** recorded for our Exp_Amb data fitted nicely to the general trend for grossulars, the parameter ***a*** for Exp_1GPa appear to be almost an outlier, although according to the confidence interval depicted in Fig. 2, such value of ***a*** is still possible to be observed in a single measurement. Note however that even for the ambient pressure measurements performed on the same instrument, a 0.01 Å deviation is observed between the high-resolution dataset used for the charge density study and the standard datasets used for the cell determination only.

4. Experimental deformation map.

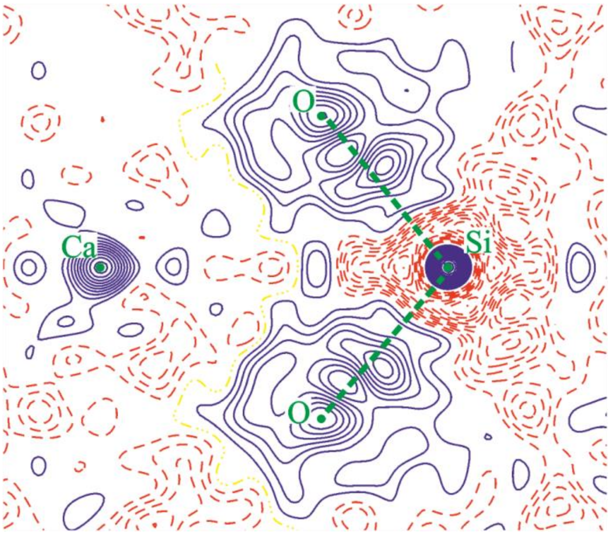

Figure 5S. Experimental deformation map (with multipolar phases). Plane Ca-Si-O.
Apart from maxima/minima at atomic positions, indicative of non-zero atomic charges, bonding density features are visible at Si – O bonds, in accordance with their covalent character. Contours at +/- 0.05eA^{-3}, blue-positive, red-negative.

5. Properties of the charge density at the (3,-1) BCPs of grossular.

Table 4S. Properties of the charge density at the (3,-1) BCPs of grossular.

Values of $d_{1\text{-bcp}}$ and $d_{2\text{-bcp}}$ denote distances from BCP to atoms 1 and 2, respectively. Values $\rho(r_c)$, $\nabla^2\rho(r_c)$, ε, λ_1, λ_2, λ_3 –stand for electron density ρ at BCP, Laplacian of ρ, ellipticity, and the Hessian tensor diagonal values. In the case of theoretical refinements (TR#i) the left and right values correspond to results from XD2016 and TOPOND14 respectively.

**X-Y interaction**		**Si-O**	**Al-O**	**Ca-O[1]**	**Ca-O[2]**
**$d_{1\text{-bcp}}$ (Å)**	Exp_Amb	0.694	0.833	1.194	1.273
	Exp_1GPa	0.696	0.853	1.191	1.266
	TR1	0.705 / 0.685	0.829 / 0.804	1.181 / 1.163	1.257 / 1.234
	TR2	0.704 / 0.685	0.827 / 0.803	1.180 / 1.162	1.254 / 1.231
	TR3	0.703 / 0.684	0.826 / 0.801	1.178 / 1.160	1.249 / 1.228
	TR4	0.703 / 0.684	0.824 / 0.800	1.176 / 1.159	1.246 / 1.225
	TR5	0.698 / 0.679	0.815 / 0.792	1.165 / 1.149	1.220 / 1.203
	Pyrope[1]	0.692	0.798	0.969	1.039
**$d_{2\text{-bcp}}$ (Å)**	Exp_Amb	0.953	1.102	1.132	1.244
	Exp_1GPa	0.957	1.089	1.143	1.231
	TR1	0.955 / 0.974	1.095 / 1.120	1.151 / 1.165	1.237 / 1.257
	TR2	0.954 / 0.973	1.093 / 1.117	1.150 / 1.164	1.233 / 1.253
	TR3	0.953 / 0.971	1.090 / 1.114	1.148 / 1.162	1.228 / 1.247
	TR4	0.951 / 0.970	1.088 / 1.112	1.147 / 1.159	1.224 / 1.243
	TR5	0.942 / 0.960	1.070 / 1.093	1.131 / 1.143	1.191 / 1.206
	Pyrope[1]	0.943	1.086	1.228	1.294
**$\rho(r_c)$ (eÅ^{-3})**	Exp_Amb	1.15	0.51	0.19	0.18
	Exp_1GPa	1.06	0.53	0.25	0.19
	TR1	1.07 / 0.90	0.40 / 0.41	0.29 / 0.28	0.16 / 0.18
	TR2	1.07 / 0.91	0.40 / 0.41	0.30 / 0.28	0.16 / 0.18
	TR3	1.08 / 0.91	0.41 / 0.41	0.30 / 0.28	0.16 / 0.18
	TR4	1.09 / 0.92	0.42 / 0.42	0.30 / 0.28	0.17 / 0.18
	TR5	1.12 / 0.95	0.44 / 0.45	0.32 / 0.30	0.19 / 0.21
	Pyrope[1]	0.89	0.49	0.27	0.21
**$\nabla^2\rho(r_c)$ (eÅ^{-5})**	Exp_Amb	8.5	3.3	5.1	2.7
	Exp_1GPa	9.2	1.0	4.4	2.8
	TR1	6.0 / 17.2	5.0 / 8.0	4.2 / 5.1	3.3 / 3.4
	TR2	6.0 / 17.4	5.0 / 8.2	4.2 / 5.1	3.4 / 3.5
	TR3	6.0 / 17.6	5.0 / 8.3	4.2 / 5,2	3.4 / 3,6
	TR4	6.0 / 17.7	5.1 / 8.4	4.3 / 5.2	3.5 / 3.6
	TR5	6.8 / 19.0	6.0 / 9,3	4.6 / 5.6	4.1 / 4.2
	Pyrope[1]	17.0	8.3	3.1	1.8
**λ_1 (eÅ^{-5})**	Exp_Amb	-8.160	-2.511	-1.119	-0.635
	Exp_1GPa	-7.551	-2.963	-1.138	-0.588
	TR1	-7.833 / -5.543	-2.899 / -2.265	-1.279 / -1.205	-0.564 / -0.699
	TR2	-7.868 / -5.567	-2.945 / -2.289	-1.282 / -1.205	-0.576 / -0.723
	TR3	-7.916 / -5.615	-3.070 / -2.338	-1.315 / -1.229	-0.598 / -0.723
	TR4	-7.938 / -5.639	-3.156 / -2.362	-1.336 / -1.229	-0.615 / -0.747
	TR5	-8.383 / -5.904	-3.302 / -2.579	-1.425 / -1.350	-0.744 / -0.892
	Pyrope[1]	-3.96	-1.61	-0.72	-0.46

**λ_2 (eÅ^{-5})**	Exp_Amb	-6.167	-2.024	-0.844	-0.504
	Exp_1GPa	-7.169	-2.913	-0.997	-0.456
	TR1	-7.409 / -5.446	-2.838 / -2.241	-1.079 / -1.181	-0.419 / -0.675
	TR2	-7.466 / -5.470	-2.873 / -2.265	-1.082 / -1.181	-0.433 / -0.699
	TR3	-7.653 / -5.519	-3.003 / -2.289	-1.098 / -1.205	-0.451 / -0.723
	TR4	-7.746 / -5.543	-3.031 / -2.313	-1.111 / -1.229	-0.467 / -0.723
	TR5	-8.014 / -5.808	-3.260 / -2.554	-1.193 / -1.325	-0.587 / -0.868
	Pyrope[1]	3.9	-1.61	-0.61	-0.40
**λ_3 (eÅ^{-5})**	Exp_Amb	22.778	7.812	7.048	3.815
	Exp_1GPa	23.897	6.834	6.573	3.838
	TR1	21.227 / 28.196	10.709 / 12.531	6.518 / 7.519	4.271 / 4.796
	TR2	21.371 / 28.389	10.858 / 12.724	6.545 / 7.543	4.360 / 4.892
	TR3	21.607 / 28.702	11.061 / 12.917	6.631 / 7.639	4.498 / 5.013
	TR4	21.708 / 28.894	11.238 / 13.086	6.702 / 7.687	4.594 / 5.109
	TR5	23.160 / 30.726	12.581 / 14.459	7.264 / 8.266	5.450 / 5.928
	Pyrope[1]	24.9	11.5	4.41	2.63
**ε**	Exp_Amb	0.323	0.241	0.326	0.261
	Exp_1GPa	0.053	0.017	0.141	0.288
	TR1	0.057 / 0.017	0.022 / 0.016	0.186 / 0.019	0.348 / 0.026
	TR2	0.054 / 0.017	0.025 / 0.016	0.184 / 0.019	0.331 / 0.026
	TR3	0.034 / 0.017	0.022 / 0.016	0.198 / 0.019	0.325 / 0.025
	TR4	0.025 / 0.017	0.041 / 0.016	0.203 / 0.019	0.317 / 0.025
	TR5	0.046 / 0.018	0.013 / 0.015	0.194 / 0.018	0.266 / 0.021
	Pyrope[1]	0.02	0.00	0.19	0.17

6. High pressure, high resolution, single crystal X-ray diffraction data collection strategy

Table 5S. Definition of the high-pressure data collection strategy.

Run number	Chi circle position	Detector position	attenuator
1	0°	0°	No
2	0°	0°	Yes
3	0°	20°	No
4	0°	40°	No
5	30°	0°	No
6	30°	20°	No
7	30°	20°	Yes
8	30°	40°	Yes
9	45°	0°	No
10	45°	20°	No
11	45°	40°	No

7. Quality of hkl data sets and the fitted models.

7.1. Quality of the fitted models. No doubt the quality of the two measured datasets differed. As it is shown in Fig. 6S, the relative average F^2_o/F^2_c ratio as a function of data resolution (sin θ/λ) for the model obtained for the data collected with the in-house diffractometer is slightly better (smoother) than the one obtained for the synchrotron dataset.

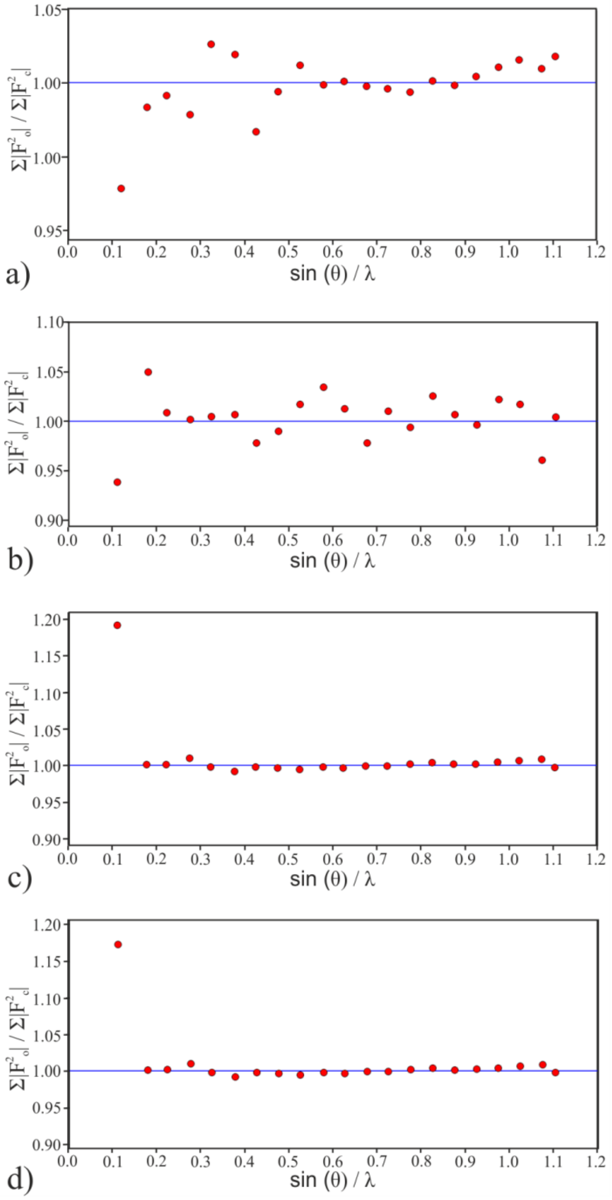

Figure 6S. Ratios of the sums of observed and calculated structure-factor amplitudes as a function of sin θ/λ. (a) Exp_Amb, (b) Exp_1GPa, (c) TR2, (d) TR1.

Fractal dimension versus residual density plots for the two datasets are displayed on Figure 7S. Although not perfect and rather broad, the fractal dimension plots are symmetric and roughly parabolic in shape, which suggests that the remaining residual density features are essentially random. One can see that peaks and holes on residual electron density map in the case of the in-house data are ca. two times smaller than in the case of the synchrotron data.

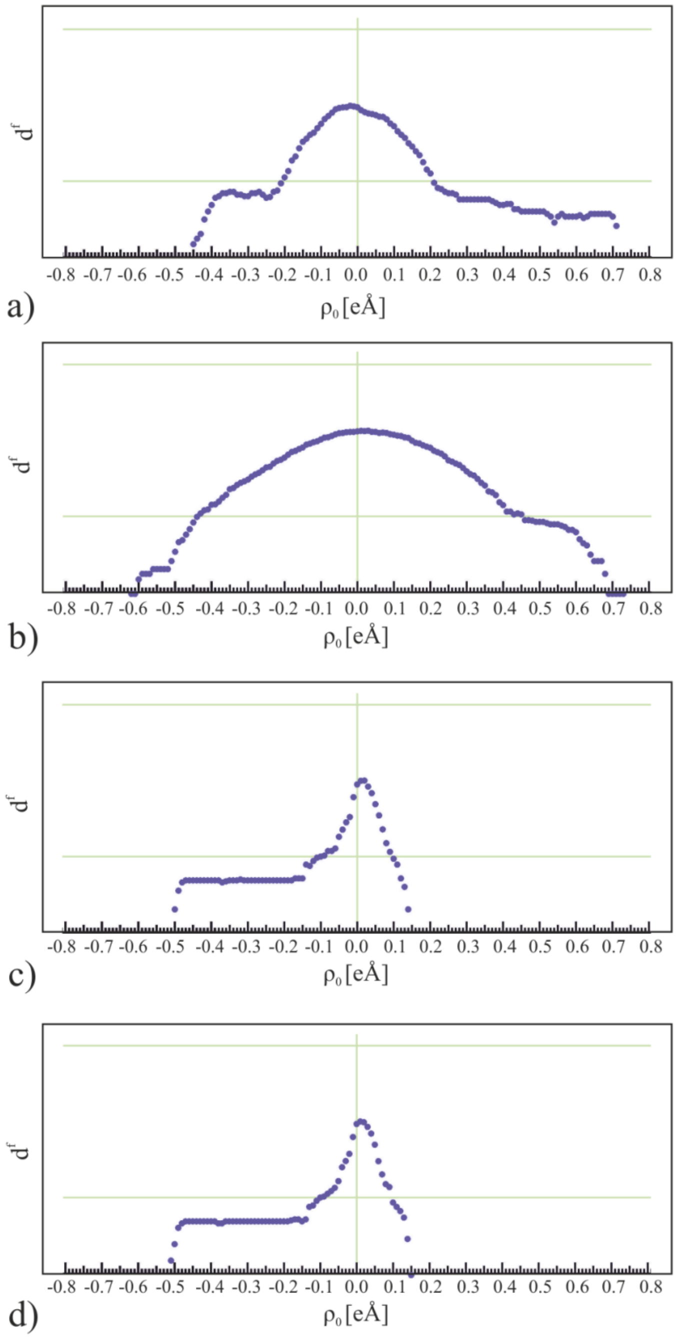

Figure 7S. Fractal dimension (d^f) vs. residual density (ρ_0).
(a) Exp_Amb, (b) Exp_1GPa, (c) TR2, (d) TR1

The large discrepancies from parabolic shape observed for fractal dimension plots of residual density maps based on theoretical data represent accumulation of two effects: firstly, the standard deviations for theoretical structure factors were assigned an arbitrary value of 1; secondly and more importantly, these feature contain discrepancies between the theoretical electron density function generated with a sophisticated basis functions and a model based on multipole model with expansion limited to hexadecapoles only.

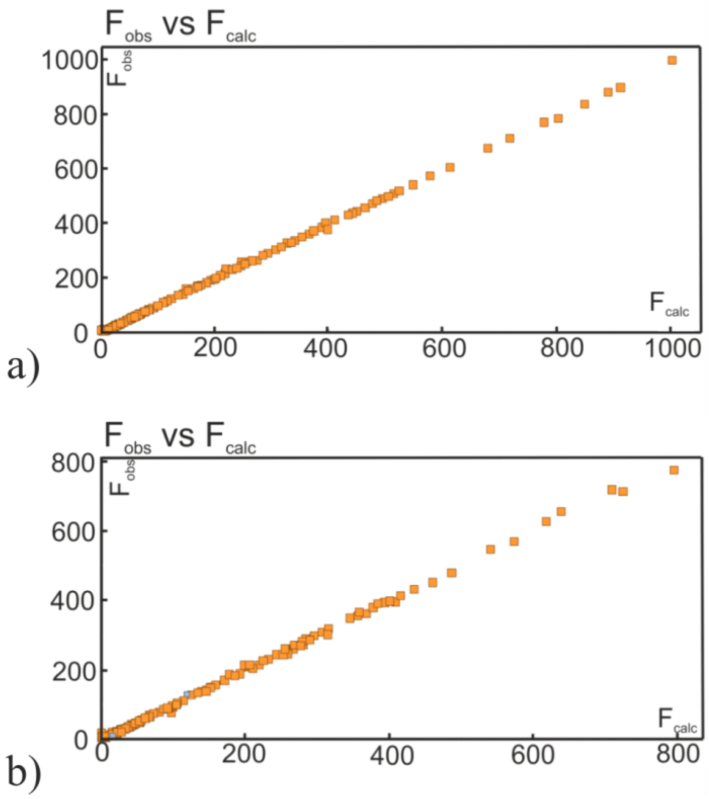

Figure 8S. F observed versus F calculated.
(a) Exp_Amb and (b) Exp_1GPa.

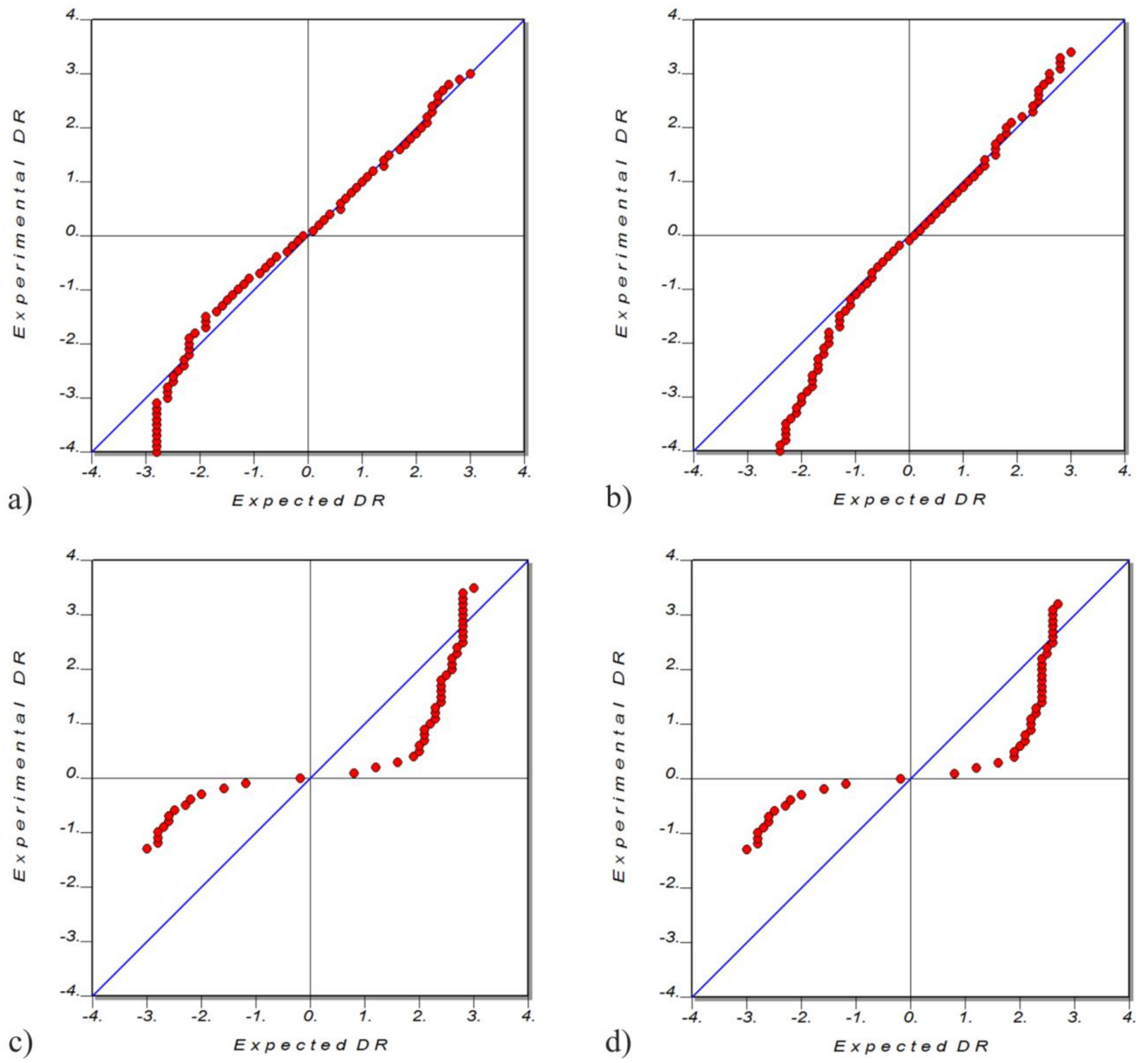

Figure 9S. Normal probability plots.
(a) Exp_Amb and (b) Exp_1GPa, (c) TR2, (d) TR1.

The shape of red plots obtained for theoretical calculations may suggest that there is a problem with standard uncertainties (s.u.). According to Henn & Meindl (Henn & Meindl, 2015) such plots are typical for situation where large s.u. values are overestimated. However, in the case of our calculations, the theoretical dynamic structure factors were generated by CRYSTAL17 without any uncertainties. To build a proper input file (hkl file) suitable to XD, for each structure factor we used an arbitrary uncertainty equal to 1. To be able to use all reflections from the list in multipole refinement the default rejection criteria in XD connected values of observed reflection with its uncertainty were disabled.

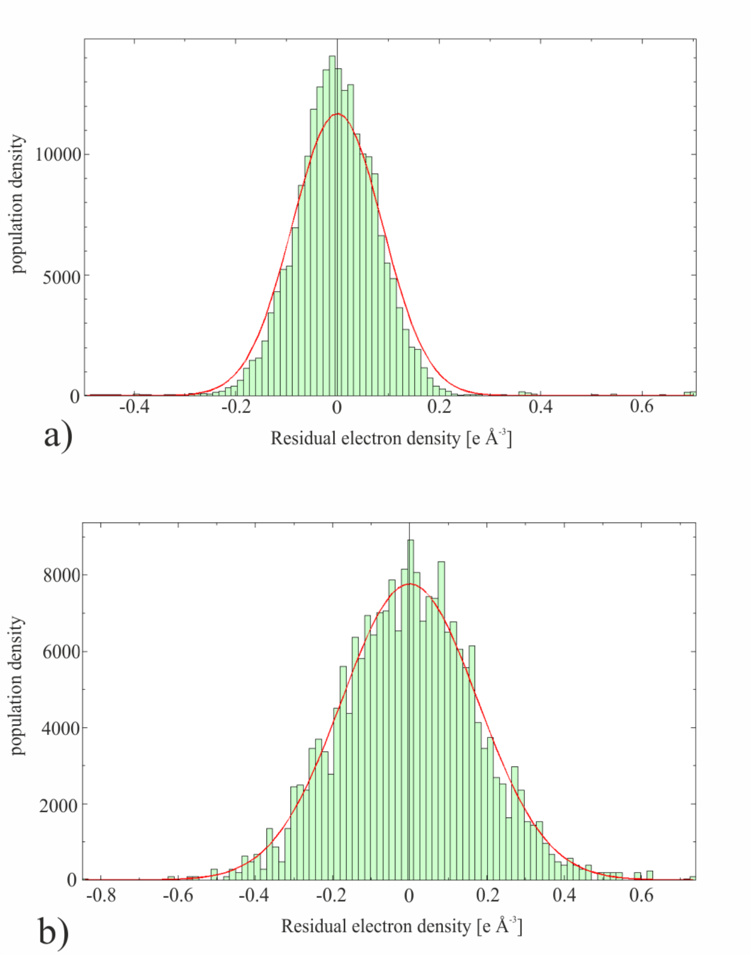

Figure 10S. Normal distribution of residual electron density.
(a) Exp_Amb and (b) Exp_1GPa.

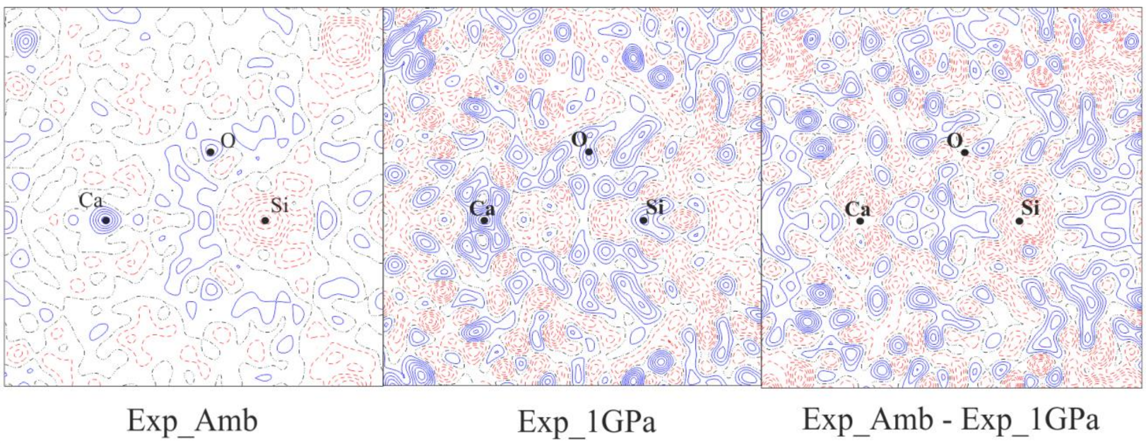

Figure 11S. Residual electron density maps. Value of contour line is 0.1e/$Å^3$. Blue and red contours correspond to positive and negative values respectively.

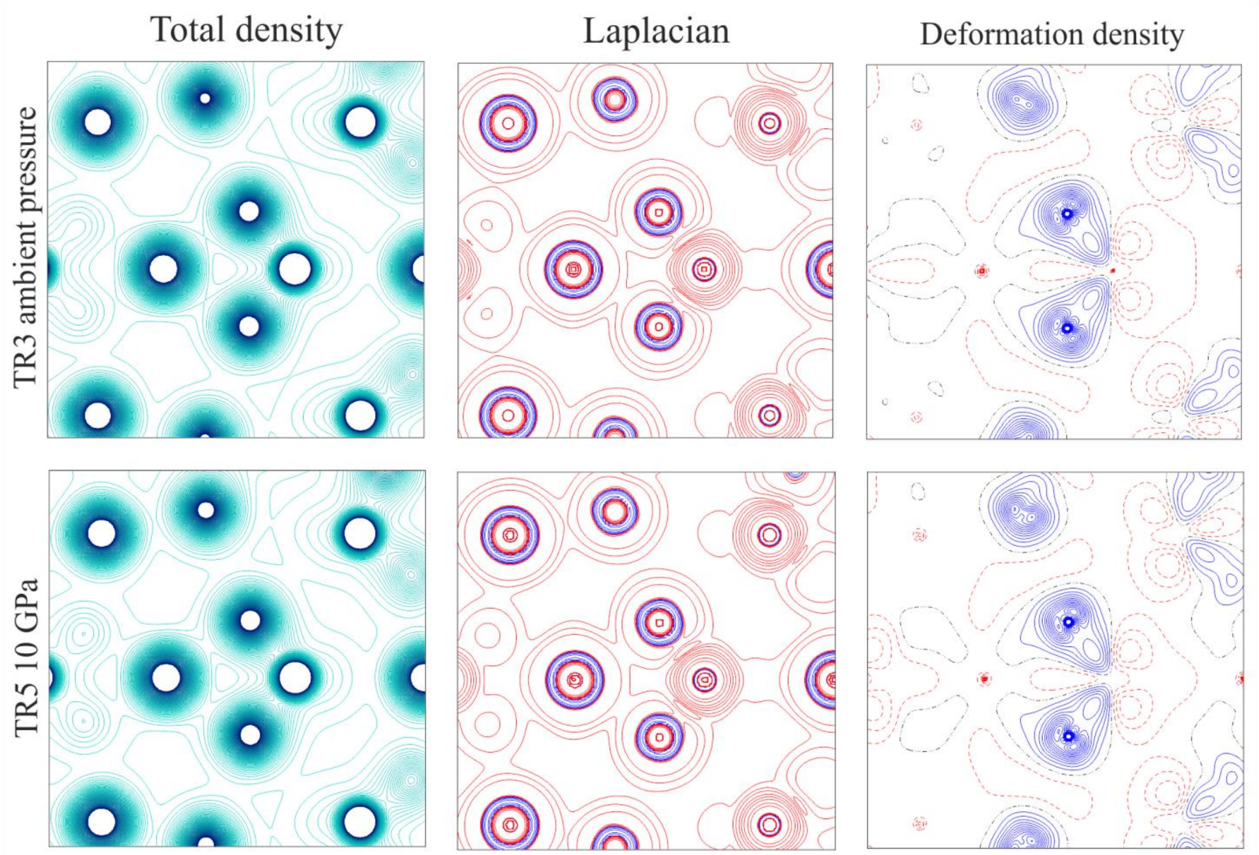

Figure 12S. Comparison of theoretical calculations for different pressures. First row - ambient pressure, second row - 10 GPa.

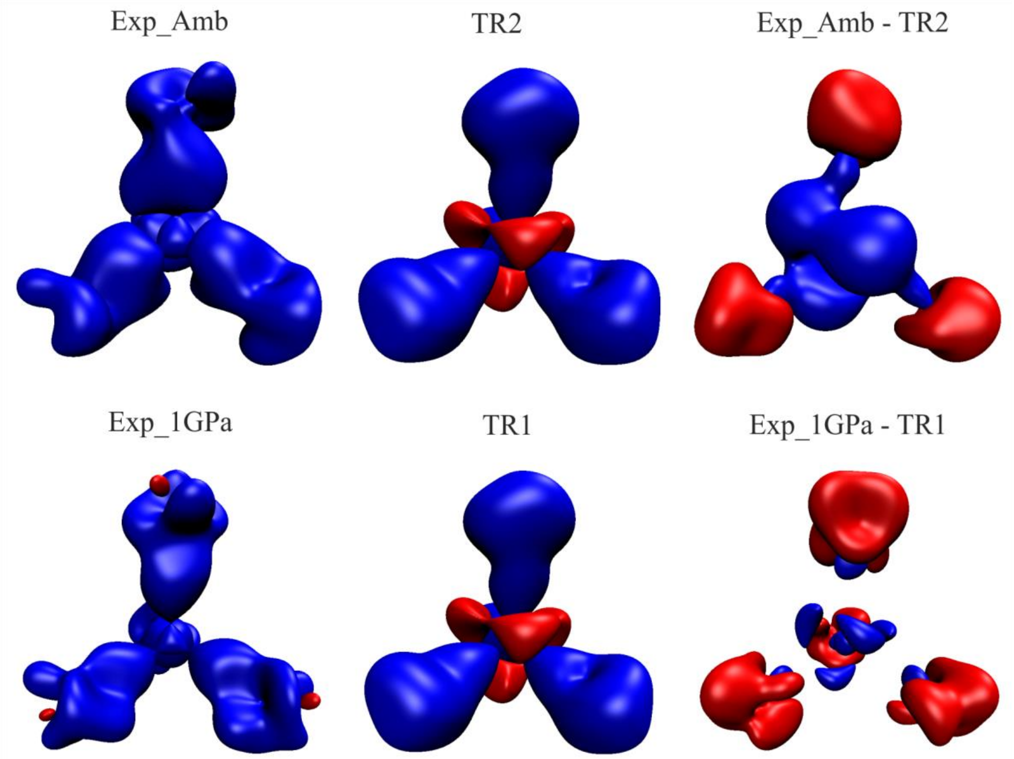

Figure 13S. 3D deformation electron maps of SiO_4. First row from left to right: experimental ambient pressure (**Exp_Amb)**, corresponding theoretical calculations (**TR2)**, difference map between the experimental and simulated maps. Second row from left to right: experimental high pressure (**Exp_1GPa)**, corresponding theoretical calculations **(TR1)**, difference **Exp_1GPa – TR1**. Blue color +0.1 $eÅ^{-3}$ and red color -0.1 $eÅ^{-3}$.

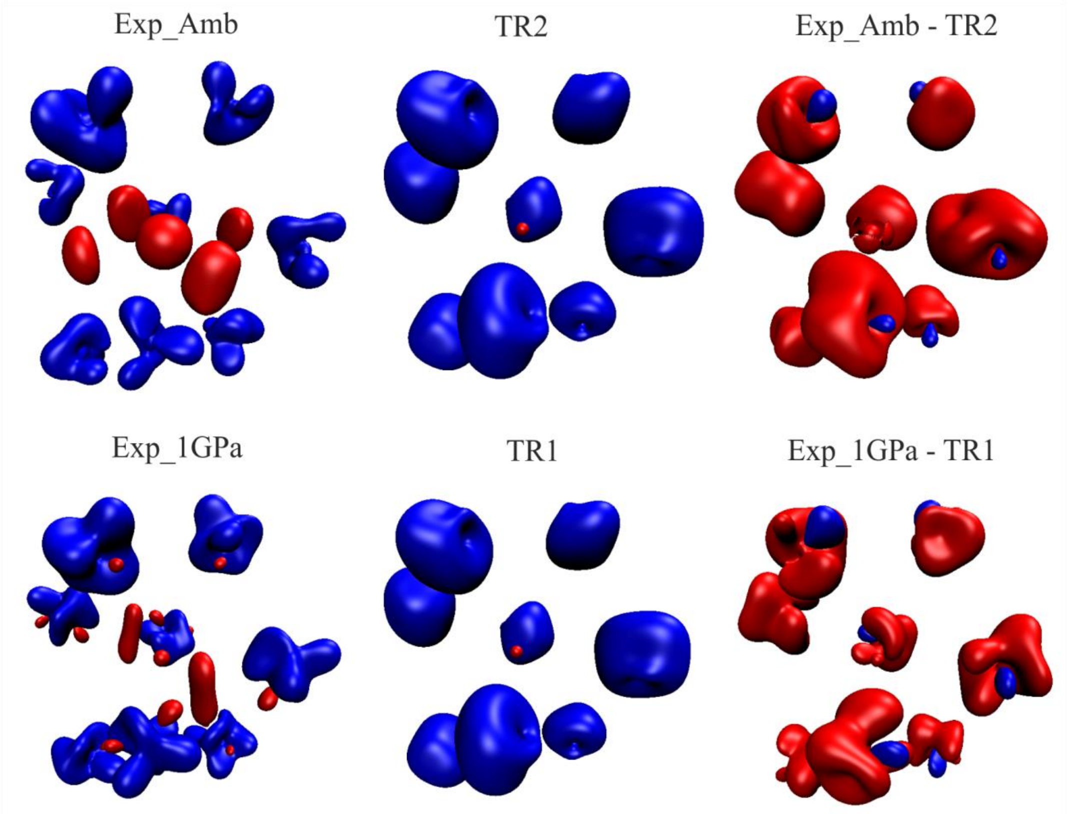

Figure 14S. 3D deformation electron density maps of CaO8. First row ambient pressure: from left to right: experimental ambient pressure (**Exp_Amb)**, corresponding theoretical calculations (**TR2)**, difference map Exp_Amb – TR2 maps. Second row from: 1 GPa left to right: experimental

high pressure (**Exp_1GPa)**, corresponding theoretical calculations **(TR1)**, difference **Exp_1GPa – TR1** maps. Blue color +0.1 e$Å^{-3}$ and red color -0.1 e$Å^{-3}$.

8. Steps of the (multipolar) refinement

The refinement strategy is detailed below, showing at each step the refined parameters. The scale factor was always refined:

1) XYX, ADPs
2) P_{val}
3) P_{val} +D
4) P_{val} +D+O
5) P_{val} +D+O+Q
6) P_{val} +D+O+Q+H
7) kappa
8) XYZ + ADPs
9) P_{val}
10) P_{val} +D
11) P_{val} +D+O
12) P_{val} +D+O+Q
13) P_{val} +D+O+Q+H

Where: P_{val}-valence electron population, D-dipole, O-octupole, Q-quadrupole, H-hexadecapole, XYZ-atomic positions. Kappa values were not refined. No significant correlation between refined parameters was observed in each cycle of refinements.

9. Theoretical results.

Table 6S. ADPs of atoms in structures of grossular obtained theoretically.

theoretical refinement 1 (1 GPa, experimental a value)						
**ADPs**	**U_{11}**	**U_{22}**	**U_{33}**	**U_{12}**	**U_{13}**	**U_{23}**
**Ca**	0.01477(2)	0.01477(2)	0.00835(3)	0.00228(3)	0	0
**Si**	0.00758(3)	0.00758(3)	0.00634(4)	0	0	0
**Al**	0.01007(3)	0.01007(3)	0.01007(3)	-0.00031(3)	-0.00031(3)	-0.00031(3)
**O**	0.00942(6)	0.01487(7)	0.01202(7)	0.00104(5)	0.00204(5)	-0.00069(5)
theoretical refinement 2 (Ambient pressure, experimental ***a*** value)						
**ADPs**	**U_{11}**	**U_{22}**	**U_{33}**	**U_{12}**	**U_{13}**	**U_{23}**
**Ca**	0.01537(3)	0.01537(3)	0.00828(3)	0.00277(3)	0	0
**Si**	0.00776(3)	0.00776(3)	0.00630(4)	0	0	0
**Al**	0.01003(3)	0.01003(3)	0.01003(3)	-0.00018(3)	-0.00018(3)	-0.00018(3)
**O**	0.00951(6)	0.01476(7)	0.01209(7)	0.00101(5)	0.00205(5)	-0.00070(5)
theoretical refinement 3 (ambient pressure, optimized ***a*** value)						
**ADPs**	**U_{11}**	**U_{22}**	**U_{33}**	**U_{12}**	**U_{13}**	**U_{23}**
**Ca**	0.01487(2)	0.01487(2)	0.00824(3)	0.00251(3)	0	0
**Si**	0.00770(3)	0.00770(3)	0.00631(4)	0	0	0
**Al**	0.00994(3)	0.00994(3)	0.00994(3)	-0.00016(3)	-0.00016(3)	-0.00016(3)
**O**	0.00940(6)	0.01465(7)	0.01197(7)	0.00099(5)	0.00203(5)	-0.00072(5)
theoretical refinement 4 (1GPa, optimized ***a*** value)						
**ADPs**	**U_{11}**	**U_{22}**	**U_{33}**	**U_{12}**	**U_{13}**	**U_{23}**
**Ca**	0.01460(2)	0.01460(2)	0.00813(3)	0.00234(3)	0	0
**Si**	0.00761(3)	0.00761(3)	0.00618(4)	0	0	0
**Al**	0.00983(3)	0.00983(3)	0.00983(3)	-0.00014(3)	-0.00014(3)	-0.00014(3)
**O**	0.00932(6)	0.01441(7)	0.01190(6)	0.00099(5)	0.00202(5)	-0.00070(5)
theoretical refinement 5 (10 GPa, optimized ***a*** value)						
**ADPs**	**U_{11}**	**U_{22}**	**U_{33}**	**U_{12}**	**U_{13}**	**U_{23}**
**Ca**	0.01265(2)	0.01265(2)	0.00752(3)	0.00134(3)	0	0
**Si**	0.00722(3)	0.00722(3)	0.00599(4)	0	0	0
**Al**	0.00896(3)	0.00896(3)	0.00896(3)	-0.00005(3)	-0.00005(3)	-0.00005(3)
**O**	0.00870(5)	0.01323(6)	0.01129(6)	0.00093(5)	0.00189(5)	-0.00063(5)

10. References

Abrahams, S. & Geller, S. (1958), *Acta Crystallographica*, **11**, 437–441.

Antao, S. M. (2013**),** *Phys. Chem. Miner.*, **40**, 705–716.

Boettcher, A. L. (**1970**), *J. Petrology*, **11**, 337–379.

Conrad, P. G., Zha, C. S., Mao, H. K. & Hemley, R. J. (**1999**),*Am. Miner.*, **84**, 374–383.

DArco, P., Fava, F. F., Dovesi, R. & Saunders, V. R. (**1996**), *J. Phys.-Condes. Matter*, **8**, 8815–8828.

Erba, A., Mahmoud, A., Belmonte, D. & Dovesi, R. (**2014),** *J. Chem. Phys.*, **140**, 124703.

Etschmann, B., Streltsov, V., Ishizawa, N. & Maslen, E. N. (**2001**), *Acta Cryst. B*, **57**, 136–141.

Ganguly, J., Cheng, W. & Oneill, H. (**1993**), *Am. Miner.*, **78**, 583–593.

Geiger, C. A. & Armbruster, T. (**1997),** *Am. Miner.*, **82**, 740–747.

Greaux, S., Kono, Y., Nishiyama, N., Kunimoto, T., Wada, K. & Irifune, T. (2011). *Phys Chem Minerals*, **38**, 85–94.

Greaux, S., Andrault, D., Gautron, L., Bolfan N. & Mezouar, M. (2014), *Phys Chem Minerals*, **41**, 419–429.

Hazen, R. M. & Finger, L. W. (1978). *American Mineralogist*, **63,** 297-303.

Henn, J. & Meindl, K. (2015). *International Journal of Materials Chemistry and Physics*, **1**, 417-430.

Lager, G., Rossman, G., Rotella, F. & Schultz, A. (**1987),** *Am. Miner.*, **72**, 766–768.

Meagher, E. (**1975**), *Am. Miner.*, **60**, 218–228.

Meyer, A., Pascale, F., Zicovich-Wilson, C. M. & Dovesi, R. (**2010),** *Int. J. Quantum Chem.*, **110**, 338–351.

Nobes, R. H., Akhmatskaya, E. V., Milman, V., Winkler, B. & Pickard, C. J. (**2000),** *Comput. Mater. Sci.*, **17**, 141–145.

Oberti, R., Quartieri, S., Dalconi, M.C., Boscherini, F., Iezzi, G., Boiocchi, M. & Eeckhout, S.G. (2006). *Am. Miner.*, **91**, 1230–1239.

Ottonello, G., Bokreta, M. & Sciuto, P. F. (**1996**), *Am. Miner.*, **81**, 429–447.

Pavese, A., Diella, V., Pischedda, V., Merli, M., Bocchio, R. & Mezouar, M. (2001). *Phys Chem Minerals*, **28**, 242-248.

Prandl, W. (**1966**), *Zeitschrift Fur Kristallographie Kristallgeometrie Kristallphysik Kristallchemie*, **123**, 81-&.

Rodehorst, U., Geiger, C. A. & Armbruster, T. (**2002**), *Am. Miner.*, **87**, 542–549.

Sawada, H. (1997a), *J. Solid state Chem.*, **132**, 300-307.

Sawada, H. (1997b), *J. Solid state Chem.*, **132**, 432-433.

Sawada, H. (1997c), *J. Solid state Chem.*, **134**, 182-186.

Sawada, H. (1999), *J. Solid state Chem.*, **142**, 273-278.

Thirumalaisamy, T. K., Saravanakumar, S., Butkute, S., Kareiva, A. & Saravanam, R. (2016), *J. Mater. Sci.-Mater. Electron.*, **27**, 1920-1928.

Zhang, L., Ahsbahs, H., Kutoglu, A. & Geiger, C. A. (1999*), Phys. Chem. Miner.*, **27**, 52-58.
